# Analysis of Selected Toll-like Receptors in the Pathogenesis and Advancement of Non-Small-Cell Lung Cancer

**DOI:** 10.3390/jcm13102793

**Published:** 2024-05-09

**Authors:** Jolanta Smok-Kalwat, Paulina Mertowska, Sebastian Mertowski, Stanisław Góźdź, Izabela Korona-Głowniak, Wojciech Kwaśniewski, Ewelina Grywalska

**Affiliations:** 1 Department of Clinical Oncology, Holy Cross Cancer Centre, 3 Artwinskiego Street, 25-734 Kielce, Poland; jolantasm@onkol.kielce.pl (J.S.-K.); stanislawgo@onkol.kielce.pl (S.G.); 2 Department of Experimental Immunology, Medical University of Lublin, 4a Chodzki Street, 20-093 Lublin, Poland; sebastianmertowski@umlub.pl (S.M.); ewelina.grywalska@umlub.pl (E.G.); 3 Institute of Medical Science, Collegium Medicum, Jan Kochanowski University of Kielce, IX Wieków Kielc 19A, 25-317 Kielce, Poland; 4 Department of Pharmaceutical Microbiology, Medical University of Lublin, 20-093 Lublin, Poland; izabela.korona-glowniak@umlub.pl; 5 Department of Gynecologic Oncology and Gynecology, Medical University of Lublin, Staszica 16 Street, 20-081 Lublin, Poland; wojciech.kwasniewski@umlub.pl

**Keywords:** toll-like receptors, non-small-cell lung cancer, biomarkers, tumor progression, innate immune system, clinicopathological characteristics

## Abstract

(1) **Background:** Non-small-cell lung cancer (NSCLC) represents a significant global health challenge, contributing to numerous cancer deaths. Despite advances in diagnostics and therapy, identifying reliable biomarkers for prognosis and therapeutic stratification remains difficult. Toll-like receptors (TLRs), crucial for innate immunity, now show potential as contributors to cancer development and progression. This study aims to investigate the role of TLR expression as potential biomarkers in the development and progression of NSCLC. (2) **Materials and Methods:** The study was conducted on 89 patients diagnosed with NSCLC and 40 healthy volunteers, for whom the prevalence of TLR2, TLR3, TLR4, TLR7, TLR8, and TLR9 was assessed on selected subpopulations of T and B lymphocytes in the peripheral blood of recruited patients along with the assessment of their serum concentration. (3) **Result:** Our study showed several significant changes in NSCLC patients at the beginning of the study. This resulted in a 5-year follow-up of changes in selected TLRs in recruited patients. Due to the high mortality rate of NSCLC patients, only 16 patients survived the 5 years. (4) **Conclusions:** The results suggest that TLRs may constitute real biomarker molecules that may be used for future prognostic purposes in NSCLC. However, further validation through prospective clinical and functional studies is necessary to confirm their clinical utility. These conclusions may lead to better risk stratification and tailored interventions, benefiting NSCLC patients and bringing medicine closer to precision.

## 1. Introduction

Non-small-cell lung cancer (NSCLC) remains a significant global health burden, representing a substantial portion of cancer-related mortalities worldwide [1,2,3]. Despite advancements in early detection and treatment strategies, the prognosis for NSCLC patients remains diverse, necessitating the identification of robust predictive biomarkers to improve clinical management and patient outcomes [4,5]. Toll-like receptors (TLRs), as key components of the innate immune system, have recently emerged as potential contributors to the complex interplay between the tumor microenvironment and cancer progression [6,7,8,9].

**Figure 1:**
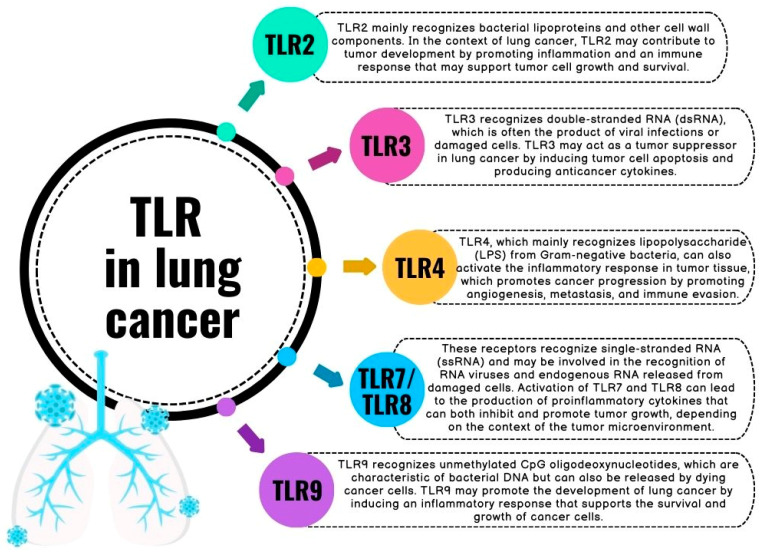
Description of the role of some TLRs in the context of lung cancer (based on [10,11,12,13,14,15,16,17,18,19,20,21]).

The innate immune system plays a fundamental role in recognizing and responding to various pathogens, and danger signals through pattern recognition receptors (PRRs), among which TLRs are prominent members [10,11]. Aside from their role in infection control, TLRs have gained considerable attention due to their involvement in tumorigenesis and tumor progression [12]. Preclinical studies have revealed that aberrant TLR signaling can promote tumor cell proliferation, metastasis, and immune evasion, implicating TLRs as potential players in the intricate network of tumorigenic processes [13,14,15,16]. TLRs play a key role in the development and treatment of lung cancer, although their functions may be opposing (Figure 1). These receptors are widely expressed on various cell types, including those in the lung epithelium as well as immune cells such as myeloid and lymphoid cells. Studies indicate that TLR3 can promote the formation of metastases but also participate in the induction of apoptosis and the reactivation of local innate reactions. Another example is the role of TLR9, the increased expression of which in tissue supported the progression and metastasis of lung cancer, and on the other hand, its activation by CpG-ODN induced anticancer effects. Activation of TLR receptors may influence the development of the immune response and the balance in the tumor microenvironment (TME), which ultimately determines its progression or regression [17,18,19,20,21].

In recent years, investigations exploring TLRs’ expression and functional significance in NSCLC have intensified. These studies have reported diverse TLR expression patterns across different NSCLC subtypes, prompting the question of whether TLRs could serve as valuable biomarkers for this heterogeneous disease [22,23,24,25]. Understanding the potential of TLR expression as an independent prognostic biomarker is crucial, as it could contribute to refining risk stratification and guiding personalized treatment approaches.

In light of the information and observations of our research team presented in the introduction, the publication aimed to determine the expression level of selected TLR receptors (TLR2, TLR3, TLR4, TLR7, TLR8, and TLR9) in subpopulations of peripheral blood lymphocytes in patients diagnosed with NSCLC. Additionally, we would like to check whether the percentage of lymphocytes positively expressing the tested TLRs and their soluble forms in serum can correlate with the patient’s survival rate. 

## 2. Materials and Methods

### 2.1. Characteristics of Patients and Research Material

Eighty-nine patients diagnosed with NSCLC and 40 healthy volunteers were analyzed. Patients were subject to several inclusion and exclusion criteria. The inclusion criteria for patients in the study included: histopathological confirmation of NSCLC; expressing the patient’s informed consent to participate in the study; and lack of treatment before starting the study. The patients had not been previously treated for lung cancer and had not received any chemotherapy, radiotherapy, and/or immunotherapy. Blood samples were obtained from previously untreated patients with suspected lung cancer one day before surgery. Only patients who had NSCLC confirmed intraoperatively and in the histopathological examination following the surgery were included in the study. The control group consisted of healthy individuals matched in terms of gender and age to the study group. The health status of patients with NSCLC was confirmed by routine diagnostic tests performed during follow-up visits (at least 2 follow-up visits per year) with an internal medicine specialist and a pulmonologist. Samples for testing were collected each time during a follow-up visit. Smoking was not considered an exclusion criterion for patients in the study. The exclusion criteria for both groups were as follows: taking medications affecting the immune system, hormonal therapy, infection during the last three months before the study, any prior history of blood transfusion, autoimmune disease, cancer, allergies, and pregnancy or lactation within one year before this study. In addition, patients in each group became good in terms of age. Patients were recruited from January 2014 to January 2015, and the status—whether they are alive or not after five years, i.e., from January 2019 to January. Detailed information on the characteristics of the patients included in this study is presented in Table 1).

The research material consisted of 5 mL of peripheral blood collected in EDTA tubes (allowing for the assessment of the immunophenotype) and 5 mL of serum (allowing for the assessment of the concentration of soluble forms of the TLRs tested). The tests were each performed in two technical repetitions. The study protocol received the necessary approval from the Bioethics Committee at the esteemed Medical University of Lublin under reference number KE-0254/283/2015.

### 2.2. Immunophenotyping

**Figure 2:**
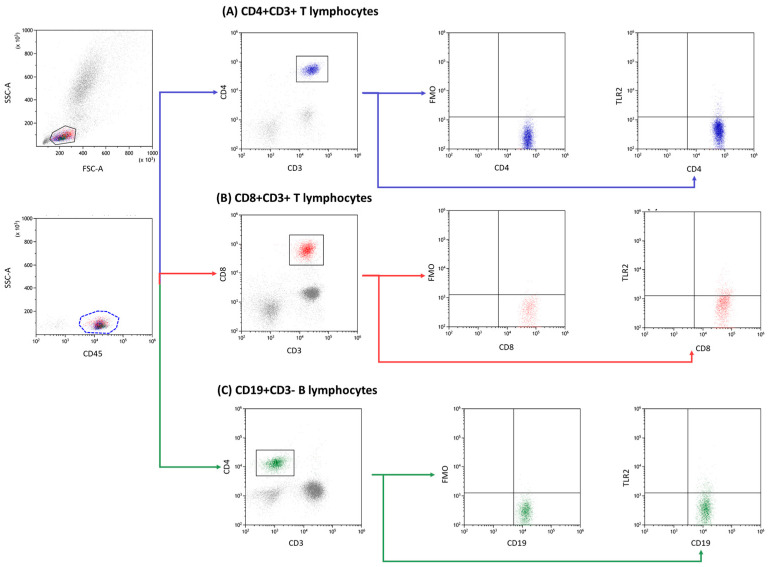
Exemplary analysis of the cells’ immunophenotype and the determination of the percentage of positive TLR expression on the example of TLRCD4+CD3+ subpopulation marked in blue, CD8+CD3+ subpopulation in red, and CD19+CD3− subpopulation in green. Points (**A**–**C**) indicate the method of reading TLR2 using the FMO control.

The analysis of lymphocyte immunophenotype in peripheral blood was performed through the use of flow cytometry, a precise and accurate approach to cell analysis. A whole blood sample was collected and treated with a set of monoclonal human anti-bodies consisting of anti-CD45 AF700, anti-CD3 PerCp, anti-CD4 BV421, anti-CD8 BV605, anti-CD19 FITC, anti-CD56 BV650, and anti-CD16 BV650, as well as anti-TLR2 APC, anti-TLR3 PE, anti-TLR4 PE, anti-TLR7 PE, anti-TLR8 APC, and anti-TLR9 APC antibodies (BioLegend, San Diego, CA 92121, USA). Subsequently, a lysing buffer was utilized to remove any red blood cells, and the remaining cells were thoroughly washed and assessed through the use of a CytoFLEX LX instrument, which is a sophisticated flow cytometer (Beckman Coulter, Indianapolis, IN, USA). The resulting data were analyzed using the Kaluza Analysis program, as demonstrated in Figure 2. The CytoFLEX LX flow cytometer was subjected to daily quality control using CytoFLEX Ready to Use Daily QC Fluorospheres reagents (Beckman Coulter, Indianapolis, IN, USA).

### 2.3. Quantification of Soluble Forms of TLR Forms

Enzyme immunoassays (ELISA) were utilized to assess the concentration of soluble forms of TLR in serum samples collected from all patients participating in our study. Commercially available kits were employed, with particular use of the Human TLR2 ELISA Kit (range: 109.4–7000 pg/mL; sensitivity 17 pg/mL), Human TLR3 ELISA Kit (range: 156–10,000 pg/mL; sensitivity 10 pg/mL), Human TLR4 ELISA Kit (range: 0.41–100 ng/mL; sensitivity 0.4 ng/well), (Abcam in Cambridge, UK,) and the Human Toll-Like Receptor 7 (TLR7) ELISA Kit (range: 10–3500 ng/L; sensitivity 5.32 ng/mL), Human Toll-Like Receptor 8 (TLR-8) ELISA Kit (range: 20–0.312 ng/mL; sensitivity 0.06 ng/mL), and Human Toll-Like Receptor 9 (TLR-9) ELISA Kit (range: 20–0.312 ng/mL; sensitivity 0.06 ng/mL) from MyBiosource in San Diego, CA, USA. The manufacturer’s instructions were followed diligently. For measurement, the VictorTM3 reader from PerkinElmer (Waltham, MA, USA).

### 2.4. Statistical Analysis 

The data generated from this study were analyzed using Tibco Statistica 13.3 software, a highly regarded platform in data analytics and visualization, based in Palo Alto, California. The normality of the data distribution was assessed using the Shapiro–Wilk test, a widely used tool for testing the normality of data. The Kruskal–Wallis test was employed to examine differences between the groups, with Dunn’s post hoc test being applied as a follow-up analysis. To account for multiple comparisons, the *p*-values for Dunn’s test were adjusted using the Bonferroni method. The study also explored the relationships between pairs of variables using Spearman’s correlation coefficients. Finally, ROC curves were utilized to evaluate the diagnostic performance of the laboratory test for patient-related parameters. To present the data clearly and concisely, GraphPad Prism, an industry-standard software platform for scientific graphing and analysis, was employed (GraphPad Prism Software v. 9.4.1, San Diego, CA, USA).

## 3. Results

### 3.1. Characteristics of Patients Included in the Study with Particular Emphasis on the Occurrence of TLR

**Figure 3:**
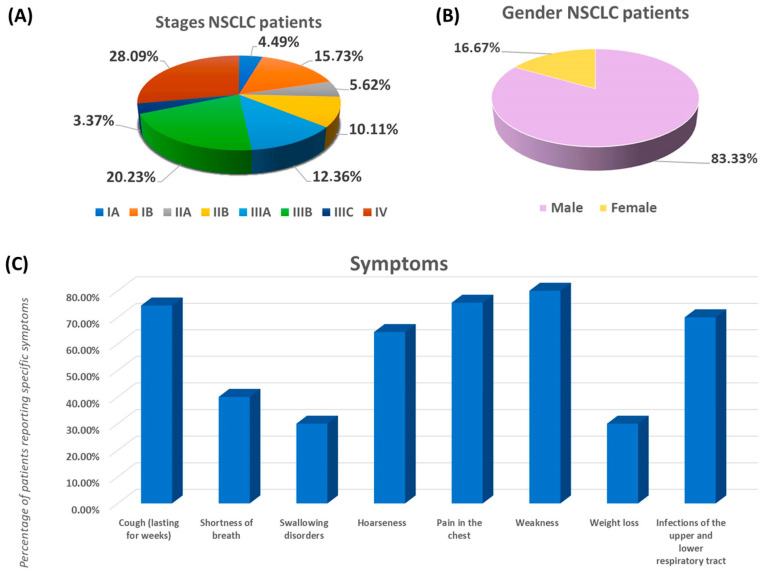
Characteristics of patients with NSCLC included in the study. (**A**) Stages of NSCLC patients; (**B**) Gender of NSCLC patients; (**C**) Most common symptoms reported by NSCLC patients.

The study recruited 89 newly diagnosed patients with a diagnosis confirmed histopathological as NSCLC. Detailed patient inclusion and exclusion criteria are described in the Materials and Methods section. The control group consisted of 40 healthy volunteers matched according to age to the study group. All collected information regarding patients recruited for the study and their results of peripheral blood morphology, biochemistry, and immunophenotype are summarized in Figure 3 and Table 2.

The average age of patients included in the study was 72 years, which corresponds to the statistical data presented by the National Health Fund for patients from Poland, for whom the average age ranges between 70 and 75 years. Of the recruited patients, the majority of patients diagnosed with NSCLC were men. Analysis of the stage of patients included in the study showed a significant percentage of patients with advanced lung cancer: 32 people in stage III, 25 people in stage IV, 14 people in stage II, 18 people in stage I. This shows the need for deeper diagnostics of this group of diseases to increase the speed and effectiveness of detection, diagnosis, and implementation of treatment for these patients. Analysis of the symptoms of patients included in the study showed that they suffered from persistent cough, hoarseness, chest pain, and general weakness. Moreover, 70% of recruited patients struggled with upper and lower respiratory tract infections requiring antibiotic therapy in the last year before diagnosis. A detailed analysis of this aspect showed that 30 people struggled with 2–3 infections during the year; 10 people had one infection during the year; no infection was recorded in another 10 people; 8 people with 4–5 infections; and 3 people reported more than 6 infections in a year. Over 97% of recruited NSCLC patients admitted to smoking cigarettes (none of the patients used electronic cigarettes). The average number of pack-years was 35.66 ± 10.03, with a median of 37.5 (minimum: 15; maximum: 60).

As we can observe in Table 2, between patients recruited for the NSCLC study and the control group, there are several statistically significant differences in the analyzed parameters of morphology, biochemistry, and immunophenotype of peripheral blood. 

However, the most important thing in our study was to determine the percentage of TLR2, TLR3, TLR4, TLR7, TLR8, and TLR9 occurrence on individual subpopulations of peripheral blood lymphocytes, the results of which are presented in Table 3.

The obtained results confirm that patients with NSCLC have a higher percentage of all TLRs tested on the tested lymphocyte subpopulations compared to healthy volunteers. We additionally confirmed this study by analyzing the level of a soluble form of TLRs (sTLRs) in the serum of all patients, and the obtained results are also presented in Table 3.

Due to the extremely interesting research data obtained, our team decided to include these patients in further studies aimed at observing changes in the levels and concentrations of TLRs over time.

### 3.2. Changes in the Percentage of TLRs and Their Concentrations in Patients with NSCLC over Time

Our goal was to monitor the same parameters for 5 years in all patients diagnosed with NSCLC. Examinations were performed routinely during follow-up visits at least once a year. Due to the extremely high compliance rate observed among lung cancer patients, our study decreased the number of patients each year. From the initial pool of 89 diagnosed patients, 25 patients died in the first year (all in stage IV); in the second year, another 18 patients (15 patients in stage IIIB and 3 patients in stage IIIC); in the third year, another 19 patients (10 stage IIIA patients and 9 stage IIB patients); in the fourth year, 3 patients (all in stage IIA). In the fifth year of our observation, 8 more patients died (4 in stage IB, 4 in stage IA). This means that of all the patients recruited for this study, only 16 patients survived the 5-year follow-up period. Detailed causes of death of patients diagnosed with NSCLC included infection in 37 people (50.00%); multi-organ failure in 20 people (27.03%); cancer cachexia (13.51%); and thromboembolic complications in 7 people (9.46%).

Each year, we compiled the results of peripheral blood morphology and biochemistry tests along with its immunophenotyping, with particular emphasis on the percentage of the tested TLRs and their serum concentration. These lists have been presented in tables and included as supplementary materials marked as Supplementary Materials Tables S1–S8). All tables take into account the division of patients into living and dead patients in a given period in which the research was carried out.

In the main part of the manuscript, we would like to focus on the last 5 years of our observations, which may highlight the importance of the role of selected TLRs as potential biomarker molecules.

**Figure 4:**
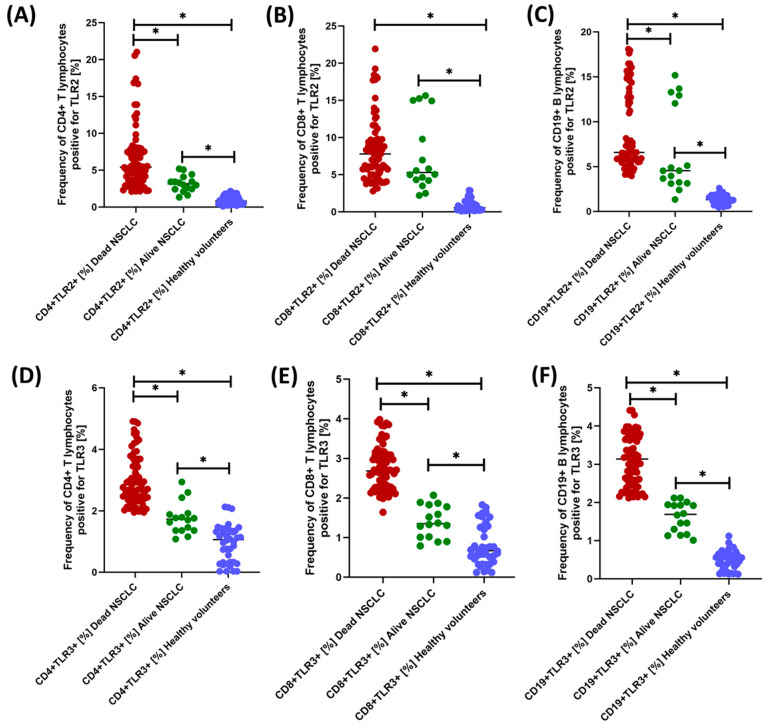
Graphical representation of the results regarding the evaluation of the percentage of TLR2- and TLR3-positive peripheral blood lymphocyte populations tested. (**A**) Percentage of CD4+TLR2+ lymphocytes; (**B**) Percentage of CD8+TLR2+ lymphocytes; (**C**) Percentage of CD19+TLR2+ lymphocytes; (**D**) Percentage of CD4+TLR3+ lymphocytes; (**E**) Percentage of CD8+TLR3+ lymphocytes; (**F**) Percentage of CD19+TLR3+ lymphocytes; * Statistically significant results are marked.

**Figure 5:**
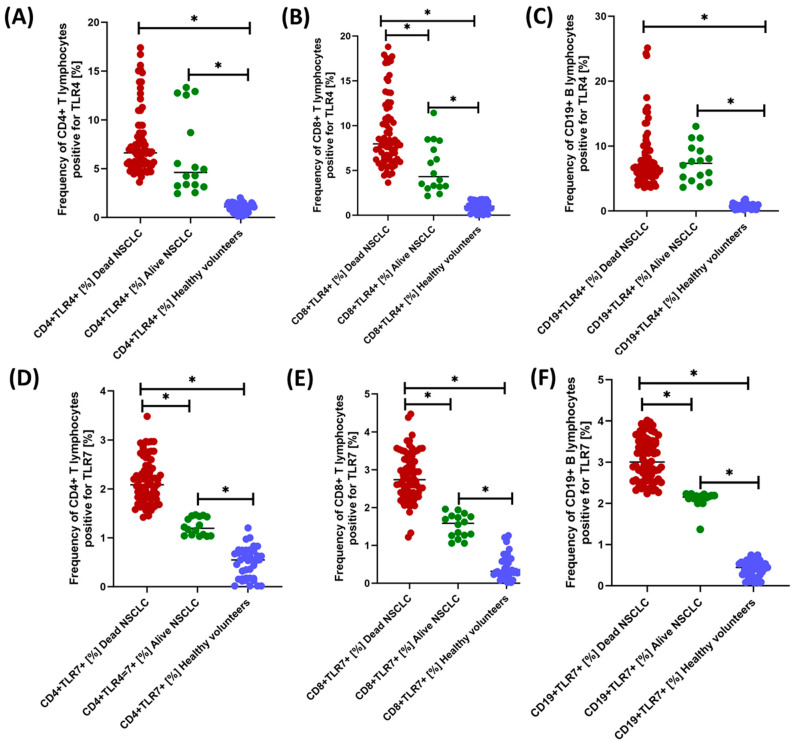
Graphical representation of the results regarding the evaluation of the percentage of TLR4- and TLR7-positive peripheral blood lymphocyte populations tested. (**A**) Percentage of CD4+TLR4+ lymphocytes; (**B**) Percentage of CD8+TLR4+ lymphocytes; (**C**) Percentage of CD19+TLR4+ lymphocytes; (**D**) Percentage of CD4+TLR7+ lymphocytes; (**E**) Percentage of CD8+TLR7+ lymphocytes; (**F**) Percentage of CD19+TLR7+ lymphocytes; * Statistically significant results are marked.

**Figure 6:**
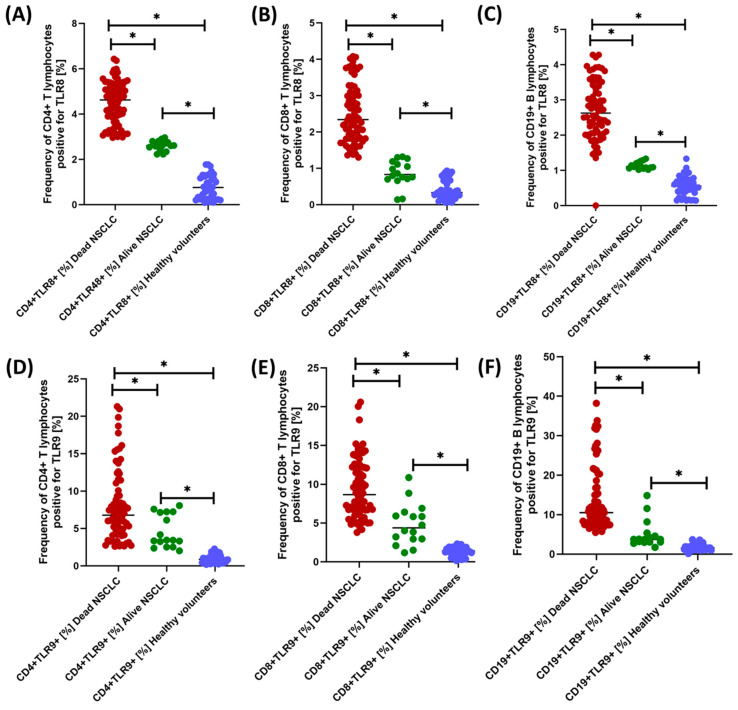
Graphical representation of the results regarding the evaluation of the percentage of TLR8- and TLR9-positive peripheral blood lymphocyte populations tested. (**A**) Percentage of CD4+TLR8+ lymphocytes; (**B**) Percentage of CD8+TLR8+ lymphocytes; (**C**) Percentage of CD19+TLR8+ lymphocytes; (**D**) Percentage of CD4+TLR9+ lymphocytes; (**E**) Percentage of CD8+TLR9+ lymphocytes; (**F**) Percentage of CD19+TLR9+ lymphocytes; * Statistically significant results are marked.

We observed much more statistically significant changes when analyzing the percentage of occurrence of individual subpopulations of T and B lymphocytes positive for the tested TLR receptors (Figure 4, Figure 5 and Figure 6 and Table 4). Of course, the level of expression of the tested TLRs was significantly higher in NSCLC patients compared to healthy volunteers, but their analysis between NSCLC alive patients and NSCLC death deserves special attention. Except for CD19+TLR4+, all median values in NSCLC death patients were higher than in NSCLC alive patients. The ratio of the recorded values ranged from 1.39-fold for CD19+TLR7+ to 2.81-fold for CD19+TLR9+, which highlights the significant range of changes in the percentage of occurrence of individual subpopulations of lymphocytes, especially B-positive for the expression of the tested TLRs.

This is also emphasized by the analyses concerning the assessment of the concentration of soluble TLRs in the serum of all tested groups of patients (Table 5). As with the immunophenotypic analyses, all observed changes between NSCLC patients and controls were statistically significant. The highest differences were observed for sTLR-9 (2.80-fold), followed by sTLR-2 (2.02-fold), sTLR-4 (1.75-fold), sTLR-8 (1.55-fold), sTLR-7 (1.36-fold), and sTLR-3 (1.20-fold) in NSCLC dead versus NSCLC alive.

Due to the high mortality of patients included in the study and their diversity in terms of stage, we decided to analyze the data obtained regarding the percentage of TLRs tested on selected T and B lymphocyte subpopulations in the context of their changes at the time of recruitment and the death of patients. For this purpose, we selected only a small group of patients with stages IA, IIIB, IIIC, and IV, because all patients did not survive the 5-year follow-up period. In the case of the remaining groups analyzed at the stage, a small percentage of patients survived the observation period; therefore, the comparison of entire groups is significantly difficult because, due to changes in the number of individual groups of patients, the obtained results may have low statistical significance. In the case of the groups selected for this analysis, detailed data were collected and are presented in tabular form (Table 6, Table 7, Table 8 and Table 9).

As we can see in Table 6, among patients with stage IA, statistically significant changes in the percentage of the tested TLRs concerned T CD8+ TLR7+ and serum concentrations of sTLR3, sTLR7, and sTLR8 between the moment of recruitment and the death of the patients. We noted much more significant correlations for stage IIIB patients, where almost all results, except the percentage of TLR9 on T and B lymphocytes and serum sTLR2 and sTLR9 concentrations, were significantly higher at the time of patients’ death than at the time of recruitment (Table 7). In patients with stages IIIC and IV, all observed changes in TLRs tested on immune cells and their serum concentrations of soluble forms were significantly higher at the time of death than at the time of recruitment of NSCLC patients to this study (Table 8 and Table 9). Due to the extremely small sample size in the relevant stages, these results should be replicated with a much larger sample size in the relevant stages. However, the results obtained from this analysis present interesting relationships that should be further explored and understood.

### 3.3. Correlation Analysis and ROC (Receiver Operating Characteristic) Curve of Dead NSCLC and Alive NSCLC Patients

**Figure 7:**
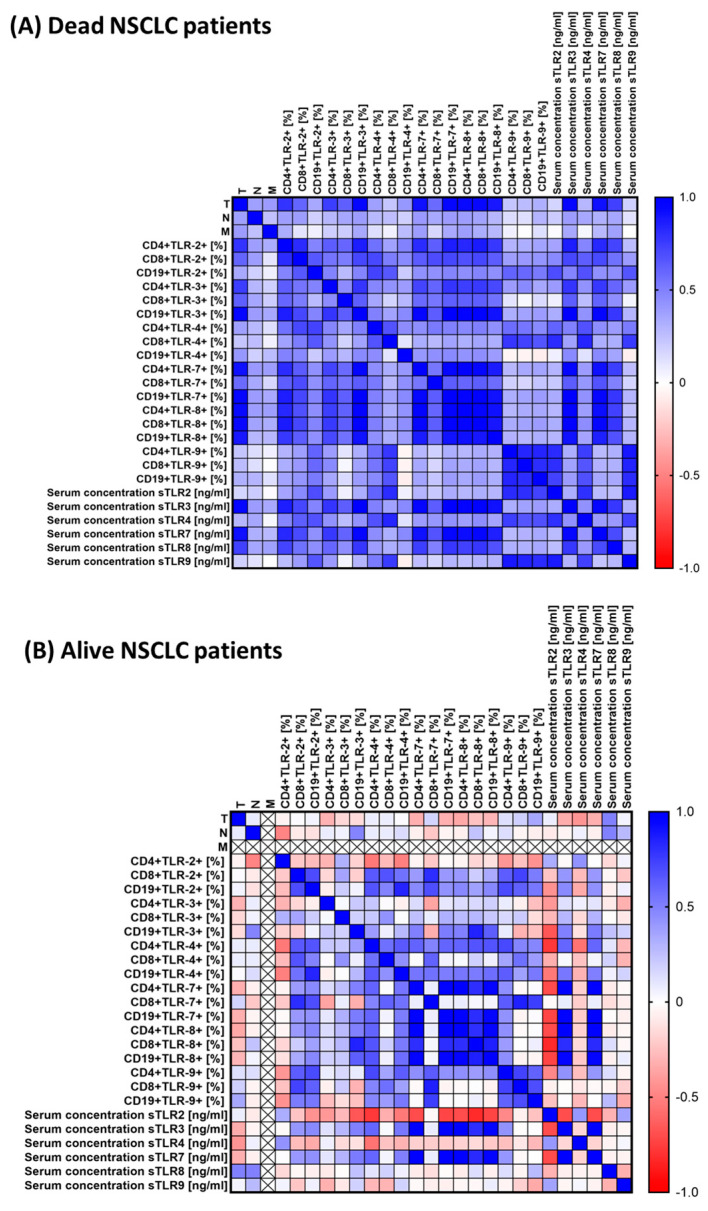
Graphical representation of Spearman rank correlations obtained for NSCLC dead (**A**) and NSCLC alive (**B**) patients. Positive correlations are marked in blue, while negative correlations are marked in red. The differentiation of shades of the mentioned colors is equivalent to the level of correlation. By positive correlations, we mean that as one parameter increases, the values of the other parameter increase, while by negative correlations we mean that as the value of one parameter increases, the values of the other parameter decrease. Abbreviations: CD—cluster of differentiation; TLR—Toll-like receptors; sTLR—soluble form of Toll-like receptors.

Next, we performed a Spearman rank correlation analysis for dead NSCLC and alive NSCLC patients. Details are provided in Supplementary Materials Tables S9 and S10, and Figure 7A,B.

In dead NSCLC patients, we can observe nearly 170 positive correlations, of which 66 were moderate, 61 were high, and 43 very high. Among the NSCLC patients alive, we recorded 80 statistically significant correlations, of which 11 were negative (1 very high, 6 high, and 4 moderate) and 69 positive (24 very high, 26 high, and 19 moderate).

**Figure 8:**
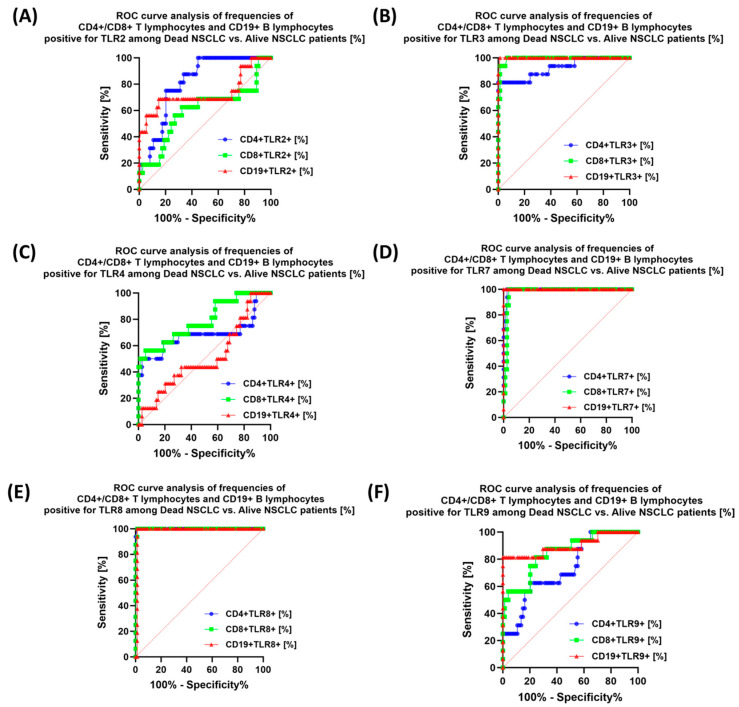
Graphical representation of the ROC analysis of selected immunophenotype parameters of dead NSCLC and alive NSCLC patients: (**A**) ROC curve for TLR2-positive lymphocyte percentage; (**B**) ROC curve for the percentage of TLR3-positive lymphocytes; (**C**) ROC curve for the percentage of TLR4-positive lymphocytes; (**D**) ROC curve for the percentage of TLR7-positive lymphocytes; (**E**) ROC curve for the percentage of TLR8-positive lymphocytes; (**F**) ROC curve for the percentage of TLR9-positive lymphocytes. Abbreviations: CD—cluster of differentiation; TLR—Toll-like receptors; ROC—Receiver Operating Characteristic.

**Figure 9:**
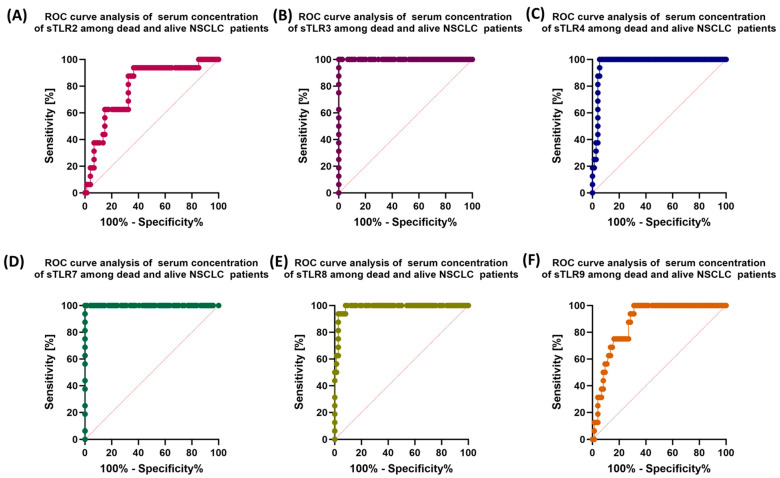
Graphical representation of ROC analysis of dissolved TLR concentrations for dead NSCLC and alive NSCLC patients: (**A**) ROC curve for sTLR2; (**B**) ROC curve for sTLR3; (**C**) ROC curve for sTLR4; (**D**) ROC curve for sTLR7; (**E**) ROC curve for sTLR8; (**F**) ROC curve for sTLR9. Abbreviations: sTLR—soluble form of Toll-like receptors; ROC—Receiver Operating Characteristic.

Due to such an important role of TLR disorders in the course of NSCLC, it seemed important to assess the prognostic value of the tested receptors in the context of mortality in NSCLC patients. The obtained test results are presented in Table 10 and Figure 7, Figure 8 and Figure 9. The most sensitive markers of poor prognosis in NSCLC patients were: sTLR3, sTLR7, and CD4+TLR8+, as well as CD19+TLR7+.

## 4. Discussion

The research results presented in this publication reflect, to some extent, the state of diagnostics and medical care not only in Poland but also around the world. The average age of patients participating in the study was 72 years, which correlates with statistical data on patients from Poland, for whom the average age is from 70 to 75 years. This indicates that the study is representative of the demographics of NSCLC patients in Poland but does not differ much from global data, where the average age of patients is over 65 years. The majority of NSCLC patients in our study were men, which is consistent with the generally higher risk of lung cancer in men. Moreover, a significant percentage of patients were in an advanced stage of the disease (32 people in stage III and 25 in stage IV), which emphasizes the need for deeper diagnostics and faster detection of this disease. Patients suffered symptoms such as chronic cough, hoarseness, chest pain, and general weakness. Additionally, 70% of patients had infections of the upper and lower respiratory tract requiring antibiotic therapy in the year preceding diagnosis, which may indicate negligence in earlier diagnosis. Virtually all patients (97%) admitted to smoking cigarettes, with an average number of pack-years of 35.66. This indicates a strong association between smoking and the development of NSCLC. Of course, this is in line with global trends, where the relationship between smoking and the development of NSCLC is well known. Moreover, studies indicate that mortality in patients who smoked was higher than mortality in never-smokers, and current smoking was an independent risk factor for worse prognosis. In our study, only three people declared that they did not smoke cigarettes (moreover, they were in IB). The expression of the tested TLRs in these patients was lower than in the remaining recruited people, but the sample size was too small to indicate statistically significant differences.

The study found that NSCLC patients had a higher prevalence of all tested TLRs (TLR2, TLR3, TLR4, TLR7, TLR8, TLR9) in peripheral blood lymphocyte subpopulations compared to healthy volunteers. This indicates a possible role of these receptors in the pathogenesis or immune response to NSCLC. Analysis of the levels of soluble forms of TLRs (sTLRs) in serum confirmed their higher concentrations in patients with NSCLC, which additionally suggests their potential importance in the dynamics of the disease. 

Our results are consistent with the literature data that confirm the involvement of TLRs in the pathogenesis of lung cancer. Much of the literature consistently shows that TLRs are expressed in NSCLC tissues, and cell lines TLR expression levels were found to be higher in NSCLC compared to healthy lung tissues. This suggests that TLRs play a role in developing and maintaining NSCLC [26,27]. TLR signaling pathways are involved in promoting cancer cell proliferation, invasion, and metastasis [8,28,29]. Activation of TLRs in NSCLC cells leads to upregulation of pro-inflammatory cytokines and chemokines (TNF-α or CCL2), contributing to the immunosuppressive nature of the tumor microenvironment [30,31,32,33,34,35,36]. TLRs (especially TLR4) have been shown to influence immune evasion mechanisms in NSCLC. Cancer cells can use TLR signaling to suppress anticancer immune responses, leading to a reduced ability of the immune system to recognize and eliminate cancer cells [37,38]. Targeting TLR signaling pathways has emerged as a potential therapeutic strategy for NSCLC. Preclinical studies have explored the use of TLR agonists and antagonists to modulate the immune response and enhance the effectiveness of treatments such as chemotherapy and immunotherapy [39,40,41,42]. Studies have shown that TLR expression profiles can influence the response to specific therapies in NSCLC. For example, TLR activation has been linked to resistance to certain chemotherapeutic agents, while TLR modulation has been shown to make cancer cells more sensitive to immunotherapies [43]. 

High expression of TLR4 has been associated with resistance to cisplatin, a commonly used chemotherapy drug in NSCLC. TLR4 activation in cancer cells can promote the upregulation of anti-apoptotic proteins and DNA repair mechanisms. leading to reduced sensitivity to cisplatin-induced cell death [44,45]. TLR7 is similarly affected, which is responsible for promoting tumor progression, resistance to chemotherapy, and, as the study indicates, poor clinical results [46]. On the other hand, TLR3 expression has been linked to increased sensitivity to chemotherapy in NSCLC. TLR3 activation in cancer cells can enhance the production of pro-apoptotic proteins and increase the susceptibility of tumor cells to chemotherapeutic agents [29,47]. Combining TLR-targeted therapies with other treatments, such as checkpoint inhibitors, has shown promising results in preclinical models. This approach aims to harness the immunomodulatory effects of TLRs to enhance the anticancer immune response and improve treatment outcomes [27,48,49,50].

The research results we presented show how the expression of the tested TLRs changes over time in individual patients depending on the stage of disease advancement. The statistically significant differences demonstrated between the percentage of tested TLRs and the concentration of their soluble forms in patients who survived the 5-year observation period were significantly lower than in patients who died. In the case of immunophenotyping tests, these differences were: 2.23-fold (CD4+TLR2+); 1.20-fold (CD8+TLR2+) and 1.37-fold (CD19+TLR2+); 1.75-fold (CD4+TLR3+); 1.92-fold (CD8+TLR3+) and 1.97-fold (CD19+TLR3+); 1.23-fold (CD4+TLR4+); 1.79-fold (CD8+TLR4+) and 1.16-fold (CD19+TLR4+); 1.82-fold (CD4+TLR7+); 1.91-fold (CD8+TLR7+) and 1.51-fold (CD19+TLR7+); 1.78-fold (CD4+TLR8+); 3.02-fold (CD8+TLR8+) and 2.5-fold (CD19+TLR8+); 1.77-fold (CD4+TLR9+); 1.97-fold (CD8+TLR9+) and 2.81-fold (CD19+TLR9+). However, in the case of serum concentrations of the tested TLRs, these differences were higher by 2.08 times (sTLR2), respectively; 1.33-fold (sTLR3); 2.32-fold (sTLR4); 1.44-fold (sTLR7); 1.63-fold (sTLR8); 2.70 times (sTLR9). Despite the limitations of this study, TLR expression on different lymphocyte subpopulations (such as CD4+, CD8+, and CD19+) may influence the way the immune system recognizes and responds to cancer cells. TLRs may promote the formation of a tumor microenvironment by inducing proinflammatory cytokines, which may support tumor cell growth and survival. Our results suggest that higher TLR expressions are associated with poorer survival, which may reflect their role in promoting cancer progression. Moreover, some TLRs can also induce apoptosis of cancer cells. Reduced TLR expression in the group of patients with better survival may indicate that, in their case, the body’s defense mechanisms were more effective in eliminating cancer cells. Additionally, as the literature data suggest, different TLRs may differentiate the immune response, e.g., by activating different types of T cells (CD4+, CD8+) and B cells (CD19+). In the case of our results, higher TLR expressions may be associated with a more aggressive or ineffective immune response, leading to worse survival outcomes. However, our studies also indicate the need for further research into the mechanisms regulating their expression and function in the context of lung cancer.

Our research is currently a pilot study and was conducted on a relatively small group of patients, which may significantly affect the aspects of TLR testing that are insufficient for clinical inclusion. However, among the obtained results, the analysis of ROC curves of NSCLC patients who did not survive the study period compared to living patients showed the highest sensitivity only for the expression of TLR7 and TLR8 on selected subpopulations of T and B lymphocytes, as well as TLR3 on B lymphocytes, in the remaining cases’ sensitivity, and the specificity of the tested TLRs was not that promising. These observations were also confirmed in the analysis of soluble forms of TLRs, for which the highest sensitivity concerned TLR3, TLR7, and TLR8. The results, although interesting and perhaps too optimistic, are only a small selection of the analyses that need to be performed to be able to include TLRs in the diagnosis of NSCLC. We hope that this research will also inspire other researchers to consider more detailed studies that will allow the involvement of TLRs as biomarker molecules in the future. 

In summary, studies on the role of TLRs in NSCLC have shown significant effects on tumor progression, immune evasion, and treatment response. The findings suggest that TLRs serve as potential prognostic markers and therapeutic targets in NSCLC, opening up new avenues for precision medicine and tailored interventions to improve patient outcomes. However further research and clinical trials are needed to confirm the clinical usefulness of TLR targeting in treating NSCLC.

## 5. Conclusions

In conclusion, this comprehensive study’s findings highlight the critical role of TLR expression in NSCLC and its potential as an independent prognostic biomarker. The analysis of NSCLC patient cohorts and healthy volunteers revealed statistically significant differences in TLR expression, indicating the involvement of TLRs in the pathogenesis of the disease. The implications of these findings are substantial, as they provide valuable insights into the complex interplay between TLR expression and NSCLC progression. Identifying potential prognostic markers holds promise for enhancing risk stratification and guiding personalized treatment approaches, ultimately leading to improved clinical management and patient outcomes.

It is important to acknowledge some limitations of this study, including the small sample size and potential confounding factors that may impact TLR expression. Thus, further validation through larger prospective studies is warranted to solidify the clinical utility of these TLR markers in NSCLC management.

Nonetheless, the results presented pave the way for future research into the molecular mechanisms underlying TLR involvement in NSCLC and open new avenues for targeted therapeutic interventions. The elucidation of TLR-related pathways and their impact on immune response and tumor microenvironment may offer novel opportunities for developing tailored immunotherapies and combination treatments.

Overall, this study contributes valuable evidence to the growing knowledge surrounding TLR expression in NSCLC and highlights its potential significance as an independent prognostic biomarker. These findings serve as a foundation for advancing precision medicine in NSCLC, aiming to improve patient stratification and treatment efficacy while fostering the development of innovative therapeutic strategies to combat this devastating disease.

## Figures and Tables

**Table 1:** Characteristics of patients included in the study.

Parameter		Patient with NSCLC (*n* = 89)		Healthy Volunteers (*n* = 40)	
		Mean ± SD	Median (Range)	Mean ± SD	Median (Range)
General information	Age	72.12 ± 8.0	73.0 (64.5–82)	74.5 ± 8.4	73.0 (70.5–83.0)
	Gender, male/female (%)	75/15 (83.33%/16.67%)		34/6 (85.0%/15.0%)	
	Smoking (%)	88 (97.77%)		14 (35.0%)	
Stages	IA	4 (4.49%)		NA	
	IB	14 (15.73%)			
	IIA	5 (5.62%)			
	IIB	9 (10.11%)			
	IIIA	11 (12.36%)			
	IIIB	18 (20.23%)			
	IIIC	3 (3.37%)			
	IV	25 (28.09%)			
Symptoms	Cough (lasting for weeks)	67 (74.44%)		NA	
	Shortness of breath	36 (40.00%)			
	Swallowing disorders	27 (30.00%)			
	Hoarseness	58 (64.44%)			
	Pain in the chest	68 (75.5%)			
	Weakness	72 (80.00%)			
	Weight loss	27 (30.00%)			
	Infections of the upper and lower respiratory tract requiring antibiotic therapy in the last year preceding diagnosis	63 (70.00%)		5 (12.5%)	
Therapy	Brachytherapy (%)	53 (58.88%)		NA	
	Chemotherapy (%)	48 (53.33%)		NA	
	Radiotherapy (%)	22 (24.44%)		NA	

NA—not applicable.

**Table 2:** Characteristics of the morphology and immunophenotype of peripheral blood of patients included in the study.

Parameter	Patient with NSCLC		Healthy Volunteers		*p*-Value
	Mean ± SD	Median (Range)	Mean ± SD	Median (Range)	
WBC [10^3^/mm^3^]	6.59 ± 2.05	6.19 (3.58–16.82)	6.29 ± 0.9	6.22 (5.6–6.8)	0.874
LYM [10^3^/mm^3^]	1.27 ± 0.54	1.15 (0.30–3.13)	1.86 ± 0.4	1.85 (1.6–2.0)	0.000 *
MON [10^3^/mm^3^]	0.55 ± 0.19	0.50 (0.23–1.18)	0.82 ± 0.3	0.77 (0.6–0.98)	0.000 *
NEU [10^3^/mm^3^]	4.59 ± 1.86	4.28 (2.18–14.76)	6.79 ± 2.9	6.55 (5.2–7.9)	0.000 *
RBC [10^6^/mm^3^]	3.12 ± 0.32	3.16 (2.24–3.74)	4.54 ± 0.3	4.55 (4.4–4.7)	0.000 *
HGB [g/gl]	9.06 ± 1.14	9.21 (6.26–12.04)	13.14 ± 1.65	13.7 (12.1–14.9)	0.000 *
PLT [10^3^/mm^3^]	228.94 ± 81.10	217.94 (84.32–540.60)	313.4 ± 93.1	312.0 (224.5–390.5)	0.000 *
CRP [mg/L]	17.54 ± 19.76	11.75 (0.50–107.63)	0.29 ± 0.4	0.16 (0.1–0.3)	0.000 *
CD3+ T lymphocytes [%]	47.47 ± 8.28	47.61 (13.87–62.55)	72.83 ± 6.4	71.94 (68.8–75.4)	0.000 *
CD3+CD8+ T lymphocytes [%]	20.38 ± 7.40	20.23 (6.09–39.93)	26.39 ± 3.0	26.91 (24.7–28.0)	0.000 *
CD3+CD4+ T lymphocytes [%]	26.63 ± 6.45	26.82 (7.05–38.76)	47.53 ± 4.8	46.76 (44.7–48.6)	0.000 *
Ratio CD3+CD4+/CD3+CD8	1.13 ± 0.96	0.92 (0.31–8.37)	1.82 ± 0.2	1.78 (1.6–2.1)	0.000 *
CD19+ B lymphocytes [%]	5.44 ± 3.07	4.91 (1.13–14.56)	12.59 ± 2.3	12.46 (11.6–13.7)	0.000 *

The symbol * denotes statistically significant results. Abbreviations: WBC—white blood cells; LYM- lymphocytes; MON—monocytes; NEU—neutrophils; RBC—red blood cells; HGB—hemoglobin; PLT—pellets; CRP—C-reactive protein; CD—cluster of differentiation.

**Table 3:** Peripheral blood immunophenotype analysis and serum concentration of sTLRs of NSCLC patients and healthy volunteers.

Lymphocyte Subset	Patient with NSCLC		Healthy Volunteers		*p*-Value
	Mean ± SD	Median (Range)	Mean ± SD	Median (Range)	
T CD4+TLR2+ [%]	4.08 ± 2.80	3.29 (0.91–14.29)	0.92 ± 0.6	0.83 (0.5–1.3)	<0.0001 *
T CD8+TLR2+ [%]	5.65 ± 3.03	4.79 (1.50–14.91)	0.88 ± 0.8	0.56 (0.3–1.2)	<0.0001 *
B CD19+TLR2+ [%]	5.78 ± 3.03	4.42 (0.90–12.29)	1.35 ± 0.5	1.32 (1.1–1.7)	<0.0001 *
T CD4+TLR3+ [%]	1.89 ± 0.61	1.79 (0.73–3.34)	0.94 ± 0.6	1.06 (0.3–1.3)	<0.0001 *
T CD8+TLR3+ [%]	1.71 ± 0.50	1.74 (0.54–2.71)	0.81 ± 0.5	0.67 (0.4–1.3)	<0.0001 *
B CD19+TLR3+ [%]	1.93 ± 0.56	1.92 (0.69–3.00)	0.49 ± 0.2	0.52 (0.3–0.6)	<0.0001 *
T CD4+TLR4+ [%]	5.06 ± 2.39	4.41 (1.67–11.83)	1.0 ± 0.5	1.08 (0.5–1.3)	<0.0001 *
T CD8+TLR4+ [%]	5.85 ± 2.76	5.24 (1.47–12.78)	0.98 ± 0.6	0.99 (0.5–1.5)	<0.0001 *
B CD19+TLR4+ [%]	5.46 ± 2.99	4.56 (2.45–17.09)	0.77 ± 0.4	0.79 (0.5–1.0)	<0.0001 *
T CD4+TLR7+ [%]	1.36 ± 0.37	1.34 (0.70–2.37)	0.49 ± 0.3	0.55 (0.2–0.7)	<0.0001 *
T CD8+TLR7+ [%]	1.76 ± 0.52	1.76 (0.72–3.04)	0.46 ± 0.3	0.32 (0.2–0.7)	<0.0001 *
B CD19+TLR7+ [%]	1.97 ± 0.41	1.94 (0.93–2.73)	0.43 ± 0.2	0.44 (0.3–0.6)	<0.0001 *
T CD4+TLR8+ [%]	2.83 ± 0.76	2.85 (1.52–4.37)	0.79 ± 0.5	0.76 (0.3–1.3)	<0.0001 *
T CD8+TLR8+ [%]	1.49 ± 0.65	1.43 (0.10–2.77)	0.42 ± 0.3	0.33 (0.3–0.7)	<0.0001 *
B CD19+TLR8+ [%]	1.66 ± 0.64	1.63 (0.69–2.91)	0.55 ± 0.3	0.56 (0.4–0.8)	<0.0001 *
T CD4+TLR9+ [%]	5.08 ± 3.10	4.31 (1.37–14.48)	0.97 ± 0.6	0.87 (0.5–1.3)	<0.0001 *
T CD8+TLR9+ [%]	5.85 ± 2.74	5.16 (0.80–14.00)	1.34 ± 0.6	1.41 (0.9–1.8)	<0.0001 *
B CD19+TLR-9+ [%]	8.25 ± 5.56	6.33 (1.18–25.96)	1.76 ± 0.7	1.59 (1.2–2.1)	<0.0001 *
sTLR2 [ng/mL]	6.78 ± 4.74	5.11 (0.80–19.35)	2.5 ± 1.0	2.63 (1.7–3.3)	<0.0001 *
sTLR3 [ng/mL]	6.25 ± 1.66	5.59 (4.79–11.89)	1.57 ± 0.8	1.47 (1.0–2.1)	<0.0001 *
sTLR4 [ng/mL]	6.34 ± 3.23	5.28 (2.14–16.02)	3.03 ± 0.7	3.26 (2.6–3.5)	<0.0001 *
sTLR7 [ng/mL]	4.92 ± 1.52	4.65 (3.47–10.10)	1.07 ± 0.6	1.07 (0.6–1.5)	<0.0001 *
sTLR8 [ng/mL]	6.22 ± 1.61	6.17 (3.49–12.14)	0.97 ± 0.6	1.01 (0.4–1.5)	<0.0001 *
sTLR9 [ng/mL]	8.82 ± 5.28	8.58 (0.88–21.56)	3.1 ± 0.6	3.21 (2.7–3.6)	<0.0001 *

The symbol * denotes statistically significant results. Abbreviations: CD—cluster of differentiation; TLR—Toll-like receptors; sTLR—soluble form of Toll-like receptors.

**Table 4:** Analysis of the percentage of TLR occurrence on selected subpopulations of peripheral blood lymphocytes in patients with NSCLC compared to healthy volunteers, with particular emphasis on survival status.

Parameter	Patient with NSCLC Alive (*n* = 16)		Patient with NSCLC Dead (*n* = 73)		Healthy Volunteers (*n* = 40)		*p*-Value	*p*-Value		
	Mean ± SD	Median (Range)	Mean ± SD	Median (Range)	Mean ± SD	Median (Range)		NSCLC Alive vs. Dead	NSCLC Alive vs. Healthy Volunteers	NSCLC Dead vs. Healthy Volunteers
T CD4+TLR2+ [%]	3.18 ± 1.1	3.16 (2.4–3.8)	7.11 ± 4.32	5.66 (2.10–21.02)	0.92 ± 0.6	0.83 (0.5–1.3)	<0.0001 *	<0.0001 *	<0.0001 *	<0.0001 *
T CD8+TLR2+ [%]	7.52 ± 4.9	5.30 (4.3–12.4)	9.02 ± 4.38	8.33 (3.17–21.93)	0.88 ± 0.8	0.56 (0.3–1.2)	<0.0001 *	0.21	<0.0001 *	<0.0001 *
B CD19+TLR2+ [%]	6.69 ± 4.8	4.55 (3.2–12.5)	9.16 ± 4.46	7.01 (3.96–18.08)	1.35 ± 0.5	1.32 (1.1–1.7)	<0.0001 *	0.0035 *	<0.0001 *	<0.0001 *
T CD4+TLR3+ [%]	1.76 ± 0.5	1.73 (1.4–1.9)	3.08 ± 0.83	2.81 (1.95–4.91)	0.94 ± 0.6	1.06 (0.3–1.3)	<0.0001 *	<0.0001 *	<0.0001 *	<0.0001 *
T CD8+TLR3+ [%]	1.40 ± 0.4	1.35 (1.0–1.8)	2.70 ± 0.54	2.77 (1.64–3.98)	0.81 ± 0.5	0.67 (0.4–1.3)	<0.0001 *	<0.0001 *	0.00016 *	<0.0001 *
B CD19+TLR3+ [%]	1.63 ± 0.4	1.69 (1.2–1.9)	3.21 ± 0.60	3.19 (2.12–4.41)	0.49 ± 0.2	0.52 (0.3–0.6)	<0.0001 *	<0.0001 *	<0.0001 *	<0.0001 *
T CD4+TLR4+ [%]	6.40 ± 4.1	4.62 (3.3–10.6)	7.91 ± 3.50	6.72 (3.62–17.39)	1.0 ± 0.5	1.08 (0.5–1.3)	<0.0001 *	0.020	<0.0001 *	<0.0001 *
T CD8+TLR4+ [%]	5.34 ± 2.8	4.30 (3.2–7.8)	9.54 ± 4.15	8.06 (3.63–18.80)	0.98 ± 0.6	0.99 (0.5–1.5)	<0.0001 *	0.0003 *	<0.0001 *	<0.0001 *
B CD19+TLR4+ [%]	7.37 ± 2.9	7.33 (4.9–9.5)	8.62 ± 4.79	6.82 (3.79–25.13)	0.77 ± 0.4	0.79 (0.5–1.0)	<0.0001 *	0.86	<0.0001 *	<0.0001 *
T CD4+TLR7+ [%]	1.23 ± 0.2	1.20 (1.1–1.4)	2.24 ± 0.44	2.19 (1.42–3.48)	0.49 ± 0.3	0.55 (0.2–0.7)	<0.0001 *	<0.0001 *	<0.0001 *	<0.0001 *
T CD8+TLR7+ [%]	1.52 ± 0.3	1.59 (1.3–1.8)	2.90 ± 0.61	2.79 (1.22–4.47)	0.46 ± 0.3	0.32 (0.2–0.7)	<0.0001 *	<0.0001 *	<0.0001 *	<0.0001 *
B CD19+TLR7+ [%]	2.09 ± 0.2	2.16 (2.1–2.2)	3.16 ± 0.48	3.22 (2.24–4.01)	0.43 ± 0.2	0.44 (0.3–0.6)	<0.0001 *	<0.0001 *	<0.0001 *	<0.0001 *
T CD4+TLR8+ [%]	2.61 ± 0.2	2.61 (2.5–2.8)	4.66 ± 0.86	4.76 (2.96–6.43)	0.79 ± 0.5	0.76 (0.3–1.3)	<0.0001 *	<0.0001 *	<0.0001 *	<0.0001 *
T CD8+TLR8+ [%]	0.86 ± 0.4	0.83 (0.7–1.2)	2.60 ± 0.75	2.40 (1.30–4.08)	0.42 ± 0.3	0.33 (0.3–0.7)	<0.0001 *	<0.0001 *	0.0004 *	<0.0001 *
B CD19+TLR8+ [%]	1.14 ± 0.1	1.11 (1.1–1.2)	2.85 ± 0.75	4.28 (2.83–1.20)	0.55 ± 0.3	0.56 (0.4–0.8)	<0.0001 *	<0.0001 *	<0.0001 *	<0.0001 *
T CD4+TLR9+ [%]	4.62 ± 2.2	3.45 (2.9–7.2)	8.16 ± 4.84	7.16 (2.60–21.30)	0.97 ± 0.6	0.87 (0.5–1.3)	<0.0001 *	0.0032 *	<0.0001 *	<0.0001 *
T CD8+TLR9+ [%]	4.82 ± 2.7	4.38 (2.9–6.2)	9.48 ± 4.04	8.44 (3.79–20.59)	1.34 ± 0.6	1.41 (0.9–1.8)	<0.0001 *	<0.0001 *	<0.0001 *	<0.0001 *
B CD19+TLR9+ [%]	5.04 ± 3.5	3.74 (3.1–5.0)	14.18 ± 8.37	11.15 (5.52–38.17)	1.76 ± 0.7	1.59 (1.2–2.1)	<0.0001 *	<0.0001 *	<0.0001 *	<0.0001 *

The symbol * denotes statistically significant results. Abbreviations: CD—cluster of differentiation; TLR—Toll-like receptors; denotes statistically significant results.

**Table 5:** The concentration of soluble forms of TLRs in the serum of NSCLC patients among healthy volunteers.

Serum Concentration [ng/mL]	Patient with NSCLC Alive (*n* = 16)		Patient with NSCLC Dead (*n* = 74)		Healthy Volunteers (*n* = 40)		*p*-Value	*p*-Value		
	Mean ± SD	Median (Range)	Mean ± SD	Median (Range)	Mean ± SD	Median (Range)		NSCLC Alive vs. Dead	NSCLC Alive vs. Healthy Volunteers	NSCLC Dead vs. Healthy Volunteers
sTLR2 [ng/mL]	5.27 ± 4.0	4.28 (3.1–6.0)	10.98 ± 7.47	8.67 (1.17–28.46)	2.5 ± 1.0	2.63 (1.7–3.3)	<0.0001 *	<0.0001 *	<0.0001 *	<0.0001 *
sTLR3 [ng/mL]	7.21 ± 0.1	7.18 (7.1–7.3)	9.62 ± 2.56	8.65 (7.3–17.48)	1.57 ± 0.8	1.47 (1.0–2.1)	<0.0001 *	<0.0001 *	<0.0001 *	<0.0001 *
sTLR4 [ng/mL]	4.47 ± 0.6	4.68 (4.2–4.8)	10.37 ± 4.79	8.63 (4.03–23.57)	3.03 ± 0.7	3.26 (2.6–3.5)	<0.0001 *	<0.0001 *	<0.0001 *	<0.0001 *
sTLR7 [ng/mL]	5.32 ± 0.2	5.25 (5.2–5.4)	7.65 ± 2.31	7.14 (5.71–14.86)	1.07 ± 0.6	1.07 (0.6–1.5)	<0.0001 *	<0.0001 *	<0.0001 *	<0.0001 *
sTLR8 [ng/mL]	6.13 ± 0.7	6.09 (5.6–6.5)	9.97 ± 2.10	9.47 (5.99–17.86)	0.97 ± 0.6	1.01 (0.4–1.5)	<0.0001 *	<0.0001 *	<0.0001 *	<0.0001 *
sTLR9 [ng/mL]	5.41 ± 2.2	5.02 (3.8–6.5)	14.61 ± 7.5	14.07 (1.30–31.70)	3.1 ± 0.6	3.21 (2.7–3.6)	<0.0001 *	<0.0001 *	<0.0001 *	<0.0001 *

The symbol * denotes statistically significant results. Abbreviations: sTLR—soluble form of Toll-like receptors.

**Table 6:** Analysis of the TLR results obtained in patients with stage IA at the time of recruitment and death.

Parameters	Results of Tested TLRs for Patients with IA at Recruitment	Results of Tested TLRs for IA Patients Who Died	*p*-Value
	Median (Range)	Median (Range)	
T CD4+TLR2+ [%]	2.48 (1.43–5.11)	2.67 (2.30–4.64)	0.885
T CD8+TLR2+ [%]	4.44 (2.45–6.59)	5.28 (2.79–5.69)	0.685
B CD19+TLR2+ [%]	4.21 (3.92–7.45)	6.55 (5.20–12.72)	0.200
T CD4+TLR3+ [%]	2.27 (2.07–2.51)	2.55 (1.95–2.95)	0.485
T CD8+TLR3+ [%]	1.96 (1.50–2.16)	2.17 (1.99–2.62)	0.200
B CD19+TLR3+ [%]	2.49 (2.45–2.54)	2.20 (2.15–2.64)	0.342
T CD4+TLR4+ [%]	3.18 (3.16–9.45)	5.66 (5.44–6.76)	0.342
T CD8+TLR4+ [%]	4.42 (2.47–11.91)	7.81 (4.77–10.01)	0.485
B CD19+TLR4+ [%]	3.93 (3.18–10.59)	4.55 (3.61–5.39)	0.885
T CD4+TLR7+ [%]	1.63 (1.57–1.79)	1.65 (1.61–1.90)	0.685
T CD8+TLR7+ [%]	1.82 (1.78–1.90)	2.08 (2.04–3.26)	0.028 *
B CD19+TLR7+ [%]	2.37 (2.28–2.40)	2.37 (2.32–2.74)	0.685
T CD4+TLR8+ [%]	3.54 (3.47–3.66)	3.13 (3.06–3.99)	0.342
T CD8+TLR8+ [%]	1.91 (1.86–2.04)	1.54 (1.39–1.97)	0.200
B CD19+TLR8+ [%]	2.32 (2.10–2.43)	1.77 (1.55–2.31)	0.114
T CD4+TLR9+ [%]	4.08 (3.22–10.42)	6.36 (5.92–17.76)	0.200
T CD8+TLR9+ [%]	6.00 (4.77–8.55)	9.75 (7.72–11.34)	0.057
B CD19+TLR9+ [%]	7.77 (7.15–9.16)	8.29 (6.58–21.26)	0.685
sTLR2 [ng/mL]	4.02 (0.80–18.33)	9.41 (7.71–14.25)	0.342
sTLR3 [ng/mL]	6.56 (6.35–7.34)	7.64 (7.56–8.09)	0.028 *
sTLR4 [ng/mL]	4.32 (2.74–8.03)	7.97 (7.23–12.79)	0.200
sTLR7 [ng/mL]	5.09 (5.05–5.14)	5.88 (5.76–6.54)	0.028 *
sTLR8 [ng/mL]	6.49 (6.26–6.68)	8.36 (7.91–11.63)	0.028 *
sTLR9 [ng/mL]	9.25 (8.21–16.46)	15.49 (6.65–19.87)	0.485

The symbol * denotes statistically significant results. Abbreviations: CD—cluster of differentiation; TLR—Toll-like receptors; sTLR—soluble form of Toll-like receptors.

**Table 7:** Analysis of the TLR results obtained in patients with stage IIIB at the time of recruitment and death.

Parameters	Results of Tested TLRs for Patients with IIIB at Recruitment	Results of Tested TLRs for IIIB Patients Who Died	*p*-Value
	Median (Range)	Median (Range)	
T CD4+TLR2+ [%]	2.73 (1.51–13.97)	5.62 (2.15–12.70)	0.001 *
T CD8+TLR2+ [%]	3.90 (2.60–9.08)	8.47 (3.17–21.93)	0.000 *
B CD19+TLR2+ [%]	4.04 (3.07–12.29)	5.87 (4.11–15.71)	0.001 *
T CD4+TLR3+ [%]	1.67 (1.33–3.34)	2.68 (2.06–4.31)	0.000 *
T CD8+TLR3+ [%]	1.71 (1.41–2.16)	2.74 (1.64–3.92)	0.000 *
B CD19+TLR3+ [%]	1.75 (1.46–3.00)	3.16 (2.12–3.93)	0.000 *
T CD4+TLR4+ [%]	3.95 (2.46–9.02)	6.55 (4.60–12.70)	0.000 *
T CD8+TLR4+ [%]	5.41 (3.02–11.59)	7.82 (5.46–17.73)	0.001 *
B CD19+TLR4+ [%]	4.04 (2.58–16.50)	6.81 (3.81–15.21)	0.000 *
T CD4+TLR7+ [%]	1.24 (0.99–2.37)	2.15 (1.42–2.88)	0.000 *
T CD8+TLR7+ [%]	1.80 (0.90–2.56)	2.85 (1.22–3.57)	0.000 *
B CD19+TLR7+ [%]	1.75 (1.54–2.70)	3.08 (2.24–3.77)	0.000 *
T CD4+TLR8+ [%]	2.55 (2.03–4.37)	4.71 (2.96–5.64)	0.000 *
T CD8+TLR8+ [%]	1.24 (0.92–2.56)	2.35 (1.30–3.79)	0.000 *
B CD19+TLR8+ [%]	1.43 (0.99–2.91)	2.64 (1.35–3.92)	0.000 *
T CD4+TLR9+ [%]	4.82 (1.77–14.26)	7.05 (2.60–14.03)	0.621
T CD8+TLR9+ [%]	5.05 (3.13–13.63)	6.91 (3.79–18.30)	0.087
B CD19+TLR9+ [%]	7.55 (4.42–22.99)	8.46 (6.54–32.35)	0.117
sTLR2 [ng/mL]	5.67 (2.60–17.69)	5.64 (2.48–25.20)	0.695
sTLR3 [ng/mL]	5.45 (5.09–11.65)	8.69 (7.48–13.49)	0.000 *
sTLR4 [ng/mL]	5.38 (3.43–12.35)	8.50 (4.21–21.46)	0.000 *
sTLR7 [ng/mL]	4.34 (3.91–10.10)	7.16 (5.71–10.56)	0.000 *
sTLR8 [ng/mL]	5.86 (4.07–10.46)	9.47 (8.41–11.92)	0.000 *
sTLR9 [ng/mL]	10.01 (3.45–19.60)	11.00 (3.33–27.83)	0.824

The symbol * denotes statistically significant results. Abbreviations: CD—cluster of differentiation; TLR—Toll-like receptors; sTLR—soluble form of Toll-like receptors.

**Table 8:** Analysis of the TLR results obtained in patients with stage IIIC at the time of recruitment and death.

Parameters	Results of Tested TLRs for Patients with IIIC at Recruitment	Results of Tested TLRs for IIIC Patients Who Died	*p*-Value
	Median (Range)	Median (Range)	
T CD4+TLR2+ [%]	3.18 (2.46–3.36)	8.35 (5.29–12.31)	0.041 *
T CD8+TLR2+ [%]	6.05 (3.39–6.38)	9.19 (8.09–11.21)	0.041 *
B CD19+TLR2+ [%]	4.10 (3.25–8.07)	14.36 (12.16–15.82)	0.021 *
T CD4+TLR3+ [%]	1.83 (1.51–1.84)	3.90 (3.09–4.25)	0.041 *
T CD8+TLR3+ [%]	1.84 (1.54–1.84)	3.09 (2.96–3.98)	0.041 *
B CD19+TLR3+ [%]	1.87 (1.64–1.90)	3.79 (2.92–3.92)	0.041 *
T CD4+TLR4+ [%]	4.91 (3.36–6.43)	8.29 (7.93–16.71)	0.041 *
T CD8+TLR4+ [%]	5.30 (4.72–9.38)	13.67 (12.58–17.27)	0.031 *
B CD19+TLR4+ [%]	4.35 (4.32–5.57)	4.26 (4.20–15.96)	0.041 *
T CD4+TLR7+ [%]	1.32 (1.22–1.35)	2.74 (2.04–2.84)	0.041 *
T CD8+TLR7+ [%]	2.23 (1.54–2.34)	3.22 (2.95–3.51)	0.041 *
B CD19+TLR7+ [%]	1.91 (1.73–1.92)	3.66 (2.88–3.76)	0.041 *
T CD4+TLR8+ [%]	2.75 (2.30–2.84)	5.43 (4.28–5.57)	0.041 *
T CD8+TLR8+ [%]	1.40 (1.18–1.41)	3.28 (2.19–3.79)	0.041 *
B CD19+TLR8+ [%]	1.60 (1.37–1.62)	3.65 (2.43–3.90)	0.041 *
T CD4+TLR9+ [%]	2.78 (2.32–6.36)	12.62 (12.43–15.69)	0.020 *
T CD8+TLR9+ [%]	4.58 (3.42–8.30)	14.05 (12.27–15.19)	0.031 *
B CD19+TLR9+ [%]	5.03 (4.67–14.59)	31.62 (15.05–32.57)	0.001 *
sTLR2 [ng/mL]	2.65 (1.93–12.05)	22.96 (20.32–24.46)	0.001 *
sTLR3 [ng/mL]	5.54 (5.39–5.56)	11.44 (8.45–13.46)	0.041 *
sTLR4 [ng/mL]	5.51 (2.86–6.08)	17.76 (17.44–18.74)	0.031 *
sTLR7 [ng/mL]	4.49 (4.25–7.18)	7.59 (6.84–7.87)	0.041 *
sTLR8 [ng/mL]	5.81 (5.75–5.86)	8.82 (8.73–11.19)	0.041 *
sTLR9 [ng/mL]	3.57 (2.72–15.90)	23.06 (22.18–25.31)	0.020 *

The symbol * denotes statistically significant results. Abbreviations: CD—cluster of differentiation; TLR—Toll-like receptors; sTLR—soluble form of Toll-like receptors.

**Table 9:** Analysis of the TLR results obtained in patients with stage IV at the time of recruitment and death.

Parameters	Results of Tested TLRs for Patients with IV at Recruitment	Results of Tested TLRs for IV Patients Who Died	*p*-Value
	Median (Range)	Median (Range)	
T CD4+TLR2+ [%]	2.03 (1.09–3.53)	7.35 (2.10–21.02)	0.000 *
T CD8+TLR2+ [%]	3.41 (1.69–10.64)	8.35 (3.61–19.25)	0.000 *
B CD19+TLR2+ [%]	3.74 (0.90–10.32)	7.33 (3.96–17.63)	0.000 *
T CD4+TLR3+ [%]	1.31 (0.73–2.05)	2.81 (1.98–4.85)	0.000 *
T CD8+TLR3+ [%]	1.24 (0.61–1.78)	2.97 (2.02–3.83)	0.000 *
B CD19+TLR3+ [%]	1.37 (0.69–1.79)	3.39 (2.15–4.41)	0.000 *
T CD4+TLR4+ [%]	3.70 (1.67–9.06)	6.95 (4.16–17.39)	0.000 *
T CD8+TLR4+ [%]	4.58 (1.47–7.77)	7.62 (4.65–18.80)	0.000 *
B CD19+TLR4+ [%]	3.74 (2.45–8.86)	6.82 (4.07–25.13)	0.000 *
T CD4+TLR7+ [%]	0.99 (0.70–1.29)	2.28 (1.56–2.97)	0.000 *
T CD8+TLR7+ [%]	1.21 (0.72–2.22)	2.66 (1.33–4.47)	0.000 *
B CD19+TLR7+ [%]	1.51 (1.36–1.86)	3.35 (2.27–4.01)	0.000 *
T CD4+TLR8+ [%]	1.91 (1.52–2.71)	5.04 (2.98–5.99)	0.000 *
T CD8+TLR8+ [%]	0.86 (0.10–1.34)	2.67 (1.36–4.08)	0.000 *
B CD19+TLR8+ [%]	0.86 (0.69–1.57)	2.98 (0.00–4.21)	0.000 *
T CD4+TLR9+ [%]	4.13 (1.37–12.08)	6.41 (2.65–21.30)	0.000 *
T CD8+TLR9+ [%]	4.36 (0.80–7.71)	8.85 (4.17–20.59)	0.000 *
B CD19+TLR9+ [%]	4.47 (1.18–14.46)	11.59 (6.50–38.17)	0.000 *
sTLR2 [ng/mL]	4.33 (1.37–13.12)	8.98 (1.17–28.46)	0.000 *
sTLR3 [ng/mL]	5.02 (4.81–5.50)	9.16 (7.49–17.48)	0.000 *
sTLR4 [ng/mL]	3.29 (2.14–8.69)	8.26 (4.03–23.57)	0.000 *
sTLR7 [ng/mL]	3.79 (3.52–4.45)	7.41 (5.75–14.86)	0.000 *
sTLR8 [ng/mL]	4.45 (3.49–7.91)	9.96 (5.99–17.04)	0.000 *
sTLR9 [ng/mL]	4.52 (2.00–13.51)	14.04 (1.30–31.70)	0.000 *

The symbol * denotes statistically significant results. Abbreviations: CD—cluster of differentiation; TLR—Toll-like receptors; sTLR—soluble form of Toll-like receptors.

**Table 10:** ROC prognostic analysis.

Factor	Parameter [%]	Prognostic Value	Youden Index	Area under the Curve (AUC)	95% CI	*p*-Value
Fatal prognosis of NSCLS patients	CD4+TLR2+ T cells [%]	5.27	0.55	0.814	0.72–0.91	<0.0001 *
	CD8+TLR2+ T cells [%]	6.03	0.3	0.60	0.43–0.77	0.26
	CD19+TLR2+ B cells [%]	5.18	0.54	0.734	0.56–0.91	0.0078 *
	CD4+TLR3+ T cells [%]	1.95	0.81	0.924	0.84–1.0	<0.0001 *
	CD8+TLR3+ T cells [%]	2.11	0.93	0.993	0.98–1.0	<0.0001 *
	CD19+TLR3+ B cells [%]	2.15	0.99	0.999	0.996–1.0	<0.0001 *
	CD4+TLR4+ T cells [%]	4.45	0.47	0.69	0.5–0.88	0.052
	CD8+TLR4+ T cells [%]	4.77	0.51	0.79	0.66–0.92	<0.0001 *
	CD19+TLR4+ B cells [%]	13.5	0.15	0.51	0.35–0.68	0.86
	CD4+TLR7+ T cells [%]	1.56	0.97	0.994	0.98–1.0	<0.0001 *
	CD8+TLR7+ T cells [%]	2.04	0.96	0.979	0.95–1.0	<0.0001 *
	CD19+TLR7+ B cells [%]	2.24	1.00	1.0	1.0	<0.0001 *
	CD4+TLR8+ T cells [%]	2.98	0.99	1.0	0.998–1.0	<0.0001 *
	CD8+TLR8+ T cells [%]	1.36	0.99	0.999	0.995–1.0	<0.0001 *
	CD19+TLR8+ B cells [%]	1.35	0.99	0.986	0.96–1.0	<0.0001 *
	CD4+TLR9+ T cells [%]	4.22	0.42	0.74	0.61–0.87	0.0003 *
	CD8+TLR9+ T cells [%]	6.69	0.57	0.85	0.74–0.95	<0.0001 *
	CD19+TLR9+ B cells [%]	5.52	0.81	0.90	0.79–1.0	<0.0001 *
	sTLR2 [ng/mL]	6.89	0.57	0.79	0.67–0.91	<0.0001 *
	sTLR3 [ng/mL]	7.48	1.00	1.0	1.0	<0.0001 *
	sTLR4 [ng/mL]	5.50	0.95	0.97	0.93–1.0	<0.0001 *
	sTLR7 [ng/mL]	5.71	1.0	1.0	1.0	<0.0001 *
	sTLR8 [ng/mL]	7.83	0.92	0.985	0.965–1.0	<0.0001 *
	sTLR9 [ng/mL]	11.46	0.69	0.87	0.80–0.95	<0.0001 *

The symbol * denotes statistically significant results.

## Data Availability

All necessary information regarding the preparation of this work is available upon written request from the corresponding author.

## Supplementary Materials

The following supporting information can be downloaded at: https://www.mdpi.com/article/10.3390/jcm13102793/s1, Table S1. Selected parameters of morphology, biochemistry, and immunophenotype of NSCLC patients after one year of observation, taking into account their survival status. Table S2. Analysis of the percentage of TLRs tested on selected subpopulations of peripheral blood lymphocytes and their serum concentration in NSCLC patients after one year of observation, taking into account their survival status. Table S3. Selected parameters of morphology, biochemistry, and immunophenotype of NSCLC patients after two years of observation, taking into account their survival status. Table S4. Analysis of the percentage of TLRs tested on selected subpopulations of peripheral blood lymphocytes and their serum concentration in NSCLC patients after two years of observation, taking into account their survival status. Table S5. Selected parameters of morphology, biochemistry, and immunophenotype of NSCLC patients after three years of observation, taking into account their survival status. Table S6. Analysis of the percentage of TLRs tested on selected subpopulations of peripheral blood lymphocytes and their serum concentration in NSCLC patients after three years of observation, taking into account their survival status. Table S7. Selected parameters of morphology, biochemistry, and immunophenotype of NSCLC patients after four years of observation, taking into account their survival status. Table S8. Analysis of the percentage of TLRs tested on selected subpopulations of peripheral blood lymphocytes and their serum concentration in NSCLC patients after four years of observation, taking into account their survival status. Table S9. Spearman rank correlation analysis for dead NSCLC patients. Table S10. Spearman rank correlation analysis for alive NSCLC patients.
