# Supplementary material for: Analysis of Selected Toll-like Receptors in the Pathogenesis and Advancement of Non-Small-Cell Lung Cancer
Source: J Clin Med. 2024 May 9;13(10):2793. doi: 10.3390/jcm13102793 (PMC11122486; doi:10.3390/jcm13102793)
Supplement: Supplementary file 1 [file jcm-13-02793-s001.zip › jcm-2984028-supplementary.pdf]

Supplementary Materials Table S1. Selected parameters of morphology, biochemistry and immunophenotype of NSCLC patients after one year of observation, taking into account their survival status

Parameters	Patients with NSCLC alive		Patients with NSCLC dead		p-value
	Mean±SD	Median (Range)	Mean±SD	Median (Range)	
WBC [$10^3/mm^3$]	6.68± 1.57	6.33 (3.90- 11.63)	11.17± 4.29	9.55 (5.44- 24.73)	0.000*
LYM [$10^3/mm^3$]	1.39± 0.55	1.25 (0.57-2.88)	1.72± 0.86	1.43 (0.44-4.60)	0.068
MON [$10^3/mm^3$]	0.57± 0.19	0.53 (0.24-1.24)	0.87± 0.30	0.79 (0.48 -1.65)	0.000*
NEU [$10^3/mm^3$]	4.45± 1.33	4.46 (2.28-8.75)	8.34± 3.87	6.84 (3.90-21.70)	0.000*
RBC [$10^6/mm^3$]	3.33± 0.33	3.36 (2.34-3.91)	4.53± 0.51	4.58 (3.60-5.36)	0.000*
HGB [g/dl]	9.76± 1.10	9.87 (7.10-12.57)	12.94± 1.91	12.90 (9.20-16.70)	
PLT [$10^3/mm^3$]	229.13± 82.06	219.04 (91.76-564.45)	386.60± 119.23	349.00 (198.00-674.00)	0.000*
CRP	15.93± 18.76	11.83 (0.53-117.13)	36.05±34.79	21.79 (1.89-134.43)	0.000*
CD3+ T cells [%]	50.72±8.59	51.62 (15.10-65.90)	70.18±13.61	69.76 (22.64-76.08)	0.000*
CD3+CD8+ T cells [%]	21.89±7.51	20.61 (7.20-41.69)	29.32±12.08	29.82 (8.95-57.23)	0.000*
CD3+CD4+ T cells [%]	28.19±6.71	28.80 (7.67-41.06)	39.80±9.85	39.66 (11.58-57.00)	0.000*
The ratio of CD3+CD4+ T cells to CD3+CD8+ T cells	1.21± 1.11	0.96 (0.33-8.74)	1.63±0.91	1.40 (0.51-4.20)	0.000*
CD19+ B cells [%]	6.47±6.68	5.57 (1.18-15.09)	8.25±5.91	6.20 (1.92-21.41)	0.265

Supplementary Materials Table S2. Analysis of the percentage of TLRs tested on selected subpopulations of peripheral blood lymphocytes and their serum concentration in NSCLC patients after one year of observation, taking into account their survival status

Parameters	Patients with NSCLC alive		Patients with NSCLC dead		p-value
	Mean±SD	Median (Range)	Mean±SD	Median (Range)	
CD4+TLR-2+ T cells [%]	3.86±2.71	3.15 (0.95-15.21)	7.85±4.70	7.35 (2.10-21.02)	0.000*
CD8+TLR-2+ T cells [%]	5.76±3.17	4.95 (1.57-16.23)	9.31±4.68	8.35 (3.61-19.25)	0.000*
CD19+TLR-2+ B cells [%]	6.03±3.34	4.54 (0.94-13.28)	9.04±4.21	7.33 (3.96-17.63)	0.000*
CD4+TLR-3+ T cells [%]	1.93±0.6	1.81 (0.77-3.63)	3.14±0.84	2.81 (1.98-4.85)	0.000*
CD8+TLR-3+ T cells [%]	1.73±0.58	1.65 (0.56-2.95)	2.86±0.49	2.97 (2.02-3.83)	0.000*
CD19+TLR-3+ B cells [%]	1.93±0.63	1.83 (0.72-3.26)	3.34±0.58	3.39 (2.15-4.41)	0.000*
CD4+TLR-4+ T cells [%]	5.16±2.40	4.34 (1.74-12.37)	8.34±3.97	6.95 (4.16-17.39)	0.000*
CD8+TLR-4+ T cells [%]	5.87±2.73	5.52 (1.53-13.12)	9.97±4.6	7.62 (4.65-18.80)	0.000*
CD19+TLR-4+ B cells [%]	5.40±2.69	4.81 (2.56-17.95)	9.60±5.67	6.82 (4.07-25.13)	0.000*
CD4+TLR-7+ T cells [%]	1.38±0.43	1.30 (0.73-2.58)	2.30±0.38	2.28 (1.56-2.97)	0.000*
CD8+TLR-7+ T cells [%]	1.81±0.61	1.78 (0.75-3.24)	2.85±0.62	2.66 (1.33-4.47)	0.000*
CD19+TLR-7+ B cells [%]	2.00±0.46	1.84 (0.97-2.94)	3.27±0.47	3.35 (2.27-4.01)	0.000*
CD4+TLR-8+ T cells [%]	2.85±0.86	2.73 (1.58-4.76)	4.80±0.80	5.04 (2.98-5.99)	0.000*
CD8+TLR-8+ T cells [%]	1.45±0.71	1.29 (0.10-3.00)	2.72±0.74	2.67 (1.36-4.08)	0.000*
CD19+TLR-8+ B cells [%]	1.66±0.72	1.49 (0.72-3.17)	2.88±0.74	2.98 (1.20-4.21)	0.000*
CD4+TLR-9+ T cells [%]	5.08±3.07	4.37 (1.43-14.89)	8.70±5.24	6.41 (2.65-21.30)	0.000*
CD8+TLR-9+ T cells [%]	5.99±3.01	5.17 (0.84-14.83)	9.53±3.79	8.85 (4.17-20.59)	0.000*
CD19+TLR-9+ B cells [%]	8.17±6.08	5.99 (1.24-25.02)	14.53±7.56	11.59 (6.50-38.17)	0.000*
The concentration of the soluble form of TLR-2 in serum [ng/mL]	6.69±4.66	4.95 (1.43-19.25)	11.97±8.12	8.98 (1.17-28.46)	0.000*
The concentration of the soluble form of TLR-3 in serum [ng/mL]	6.39±1.69	5.70 (5.00-12.68)	10.25±2.73	9.16 (7.49-17.48)	0.000*

The concentration of the soluble form of TLR-4 in serum [ng/mL]	6.45±3.44	5.48 (2.23-15.88)	10.55±4.86	8.26 (4.03-23.57)	0.000*
The concentration of the soluble form of TLR-7 in serum [ng/mL]	5.04±1.61	4.53 (3.62-11.00)	8.04±2.33	7.41 (5.75-14.86)	0.000*
The concentration of the soluble form of TLR-8 in serum [ng/mL]	6.32±1.74	6.40 (3.64-13.22)	10.31±2.21	9.96 (5.99-17.04)	0.000*
The concentration of the soluble form of TLR-9 in serum [ng/mL]	8.75±5.54	8.14 (2.09-21.33)	15.42±7.86	14.04 (1.30-31.70)	0.000*

Supplementary Materials Table S3. Selected parameters of morphology, biochemistry and immunophenotype of NSCLC patients after two years of observation, taking into account their survival status

Parameters	Patients with NSCLC alive		Patients with NSCLC dead		p-value
	Mean±SD	Median (Range)	Mean±SD	Median (Range)	
WBC [10^3/mm^3]	7.31± 1.77	7.13 (4.10-11.79)	10.47± 3.59	9.55 (5.44-24.73)	0.000*
LYM [10^3/mm^3]	1.46± 0.56	1.31 (0.63-2.99)	1.85± 0.87	1.69 (0.44-4.60)	0.02*
MON [10^3/mm^3]	0.61± 0.21	0.57 (0.27-1.36)	0.83± 0.29	0.76 (0.43-1.65)	0.000*
NEU [10^3/mm^3]	4.73± 1.41	4.72 (2.50-9.22)	7.49± 3.32	6.70 (3.54-21.70)	0.000*
RBC [10^6/mm^3]	3.60± 0.34	3.66 (2.57-4.29)	4.54± 0.49	4.56 (3.60-5.36)	0.000*
HGB [g/dl]	10.60± 1.25	10.73 (7.80-13.81)	13.03± 1.71	12.90 (9.20-16.70)	0.000*
PLT [10^3/mm^3]	242.65± 94.96	222.69 (96.72-620.10)	351.19± 93.20	347.00 (164.00577.00)	0.000*
CRP	16.07± 20.22	11.66 (0.58-123.46)	31.45± 31.16	21.54 (0.73-134.43)	0.000*
CD3+ T cells [%]	55.60±7.90	54.56 (35.25-71.74)	69.01±14.25	70.17 (20.40-76.80)	0.000*
CD3+CD8+ T cells [%]	23.37±7.94	21.98 (11.30-45.80)	29.98±11.61	30.21 (8.95-57.23)	0.000*
CD3+CD4+ T cells [%]	31.42±6.59	31.98 (20.12-43.27)	37.95±10.37	37.80 (10.37-57.00)	0.000*
Ratio of CD3+CD4+ T cells to CD3+CD8+ T cells	1.38±1.32	1.24 (0.36-9.61)	1.53±1.01	1.27 (0.51-5.58)	0.33
CD19+ B cells [%]	6.12±3.19	5.77 (1.29-15.97)	9.62±7.48	8.32 (1.92-17.00)	0.02*

Supplementary Materials Table S4. Analysis of the percentage of TLRs tested on selected subpopulations of peripheral blood lymphocytes and their serum concentration in NSCLC patients after two years of observation, taking into account their survival status

Parameters	Patients with NSCLC alive		Patients with NSCLC dead		p-value
	**Mean±SD**	**Median (Range)**	**Mean±SD**	**Median (Range)**	
CD4+TLR-2+ T cells [%]	3.74±2.94	2.85 (1.05-16.03)	7.31±4.08	6.67 (2.10-21.02)	0.000*
CD8+TLR-2+ T cells [%]	5.79±3.32	4.47 (1.72-14.37)	9.27±4.46	8.56 (3.17-21.93)	0.000*
CD19+TLR-2+ B cells [%]	6.26±3.49	5.00 (1.04-14.10)	9.02±4.38	6.74 (3.96-17.63)	0.000*
CD4+TLR-3+ T cells [%]	1.93±0.69	1.86 (0.84-3.83)	3.12±0.79	2.93 (1.98-4.85)	0.000*
CD8+TLR-3+ T cells [%]	1.66±0.51	1.69 (0.62-2.81)	2.92±0.57	2.96 (1.64-3.98)	0.000*
CD19+TLR-3+ B cells [%]	1.89±0.63	1.77 (0.79-3.44)	3.29±0.56	3.28 (2.12-4.41)	0.000*
CD4+TLR-4+ T cells [%]	5.25±2.53	4.33 (1.91-11.72)	8.21±3.63	6.95 (4.16-17.39)	0.000*
CD8+TLR-4+ T cells [%]	5.61±2.47	5.54 (1.68-13.29)	10.14±4.36	8.06 (4.65-18.80)	0.000*
CD19+TLR-4+ B cells [%]	5.66±2.83	5.00 (2.82-18.92)	8.88±4.97	6.82 (3.81-25.13)	0.000*
CD4+TLR-7+ T cells [%]	1.37±0.42	1.31 (0.80-2.71)	2.28±0.40	2.27 (1.42-2.97)	0.000*
CD8+TLR-7+ T cells [%]	1.82±0.64	1.68 (0.83-3.42)	2.86±0.60	2.78 (1.22-4.47)	0.000*
CD19+TLR-7+ B cells [%]	2.02±0.43	1.96 (1.07-3.10)	3.23±0.46	3.28 (2.24-4.01)	0.000*
CD4+TLR-8+ T cells [%]	2.81±0.84	2.52 (1.74-5.02)	4.76±0.78	4.91 (2.96-5.99)	0.000*
CD8+TLR-8+ T cells [%]	1.36±0.71	1.29 (0.11-3.17)	2.69±0.73	2.65 (1.30-4.08)	0.000*
CD19+TLR-8+ B cells [%]	1.59±0.72	1.52 (0.80-3.34)	2.89±0.73	2.92 (1.20-4.21)	0.000*
CD4+TLR-9+ T cells [%]	5.19±3.25	4.78 (1.58-16.36)	8.34±4.80	7.16 (2.60-21.30)	0.000*
CD8+TLR-9+ T cells [%]	6.02±2.94	5.55 (0.92-15.63)	9.56±4.10	8.85 (3.79-20.59)	0.000*
CD19+TLR-9+ B cells [%]	7.59±5.41	6.45 (1.36-26.37)	14.75±8.62	11.54 (6.50-38.17)	0.000*
The concentration of the soluble form of TLR-2 in serum [ng/mL]	6.43±3.89	5.48 (1.58-20.30)	11.85±8.22	8.98 (1.17-28.46)	0.000*
The concentration of the soluble form of TLR-3 in serum [ng/mL]	6.59±1.75	6.09 (5.49-13.36)	10.00±2.41	9.08 (7.48-17.48)	0.000*

The concentration of the soluble form of TLR-4 in serum [ng/mL]	6.04±3.05	5.57 (2.45-14.17)	11.03±4.98	8.81 (4.03-23.57)	0.000*
The concentration of the soluble form of TLR-7 in serum [ng/mL]	5.28±1.87	4.78 (3.98-11.59)	7.75±1.92	7.39 (5.71-14.86	0.000*
The concentration of the soluble form of TLR-8 in serum [ng/mL]	6.50±1.95	6.43 (4.00-13.93)	10.03±1.84	9.72 (5.99-17.04)	0.000*
The concentration of the soluble form of TLR-9 in serum [ng/mL]	8.75±5.26	8.88 (2.29-22.49)	14.89±8.34	14.04 (1.30-31.70)	0.000*

Supplementary Materials Table S5. Selected parameters of morphology, biochemistry and immunophenotype of NSCLC patients after three years of observation, taking into account their survival status

Parameters	Patients with NSCLC alive		Patients with NSCLC dead		p-value
	Mean±SD	Median (Range)	Mean±SD	Median (Range)	
WBC [10^3/mm^3]	7.33± 1.73	7.12 (4.45- 10.36)	10.03± 3.27	9.31 (5.26-24.73)	0.00*
LYM [10^3/mm^3]	1.69± 0.62	1.61 (0.83-3.10)	1.77± 0.79	1.62 (0.44-4.60)	0.607
MON [10^3/mm^3]	0.64± 0.23	0.60 (0.28-1.41)	0.82± 0.27	0.76 (0.41-1.65)	0.03*
NEU [10^3/mm^3]	4.78 ±1.43	4.78 (2.60-7.81)	7.17± 2.97	6.51 (3.48-21.70)	0.01*
RBC [10^6/mm^3]	3.81± 0.39	3.93 (2.67-4.46)	4.55± 0.46	4.63 (3.60- 5.36)	0.07
HGB [g/dl]	11.31± 1.48	11.30 (8.10-14.34)	13.11± 1.57	13.25 (9.20-16.70)	0.029
PLT [10^3/mm^3]	264.01±106.35	241.79 (117.45-643.95)	336.45± 104.68	329.50 (124.00-577.00)	0.01*
CRP	12.55± 12.74	8.77 (0.60-52.20)	30.47±32.04	18.98 (0.73-134.43)	0.094
CD3+ T cells [%]	59.05±8.10	48.08 (24.55-77.22)	68.87±12.93	69.94 (20.40-76.8)	0.02*
CD3+CD8+ T cells [%]	57.61±48.61	24.24 (12.01-41.25)	30.01±11.66	30.02 (8.95-58.72)	0.02*
CD3+CD4+ T cells [%]	33.55±6.94	34.74 (20.89-46.00)	38.36±9.64	38.39 (10.37-57.00)	0.098
The ratio of CD3+CD4+ T cells to CD3+CD8+ T cells	1.26±0.56	1.14 (0.53-2.95)	1.71±1.62	1.31 (0.46-5.58)	0.018
CD19+ B cells [%]	6.49±2.83	6.29	8.18±4.97	6.80	0.094

		(1.34-14.73)		(1.92-17.0)	

Supplementary Materials Table S6. Analysis of the percentage of TLRs tested on selected subpopulations of peripheral blood lymphocytes and their serum concentration in NSCLC patients after three years of observation, taking into account their survival status

Parameters	Patients with NSCLC alive		Patients with NSCLC dead		p-value
	Mean±SD	Median (Range)	Mean±SD	Median (Range)	
CD4+TLR-2+ T cells [%]	2.57±0.76	2.43 (1.15-4.20)	7.29±4.31	5.72 (2.10-21.02)	0.02*
CD8+TLR-2+ T cells [%]	5.12±3.16	4.05 (1.90-12.67)	9.23±4.34	8.46 (3.17-21.93)	0.02*
CD19+TLR-2+ B cells [%]	5.45±3.01	4.71 (1.14-12.29)	9.32±4.47	7.30 (3.96-18.08)	0.254
CD4+TLR-3+ T cells [%]	1.68±0.45	1.62 (0.93-2.53)	3.12±0.82	2.87 (1.95-4.91)	0.000*
CD8+TLR-3+ T cells [%]	1.43±0.41	1.59 (0.68-2.12)	2.85±0.53	2.84 (1.64-3.98)	0.000*
CD19+TLR-3+ B cells [%]	1.56±0.35	1.72 (0.87-2.14)	3.25±0.58	3.21 (2.12-4.41)	0.000*
CD4+TLR-4+ T cells [%]	4.98±2.49	4.42 (2.06-10.79)	8.04±3.49	6.84 (4.16-17.39)	0.104
CD8+TLR-4+ T cells [%]	5.17±2.06	5.36 (1.86-9.25)	9.63±4.16	7.96 (3.63-18.80)	0.07
CD19+TLR-4+ B cells [%]	5.28±1.99	4.64 (2.92-10.55)	8.75±4.83	6.95 (3.79-25.13)	0.01*
CD4+TLR-7+ T cells [%]	1.17±0.21	1.18 (0.89-1.54)	2.26±0.43	2.20 (1.42-3.48)	0.000*
CD8+TLR-7+ T cells [%]	1.49±0.37	1.51 (0.86-2.64)	2.93±0.60	2.88 (1.22-4.47)	0.03*
CD19+TLR-7+ B cells [%]	1.84±0.18	1.81 (1.18-2.22)	3.19±0.47	3.23 (2.24-4.01)	0.000*
CD4+TLR-8+ T cells [%]	2.37±0.30	2.31 (1.92-3.23)	4.73±0.82	4.79 (2.96-6.43)	0.000*
CD8+TLR-8+ T cells [%]	0.96±0.37	1.04 (0.12-1.60)	2.65±0.74	2.47 (1.30-4.08)	0.000*
CD19+TLR-8+ B cells [%]	1.18±0.31	1.04 (0.88-1.87)	2.90±0.74	2.84 (1.20-4.28)	0.000*
CD4+TLR-9+ T cells [%]	4.74±2.39	4.94 (1.74-14.39)	8.21±4.90	7.12 (2.60-21.30)	0.62
CD8+TLR-9+ T cells [%]	5.41±2.44	5.39 (0.96-9.91)	9.48±4.08	8.30 (3.79-20.59)	0.34
CD19+TLR-9+ B cells [%]	5.71±3.36	5.67 (1.41-17.22)	14.45±8.41	11.47 (5.52-38.17)	0.60
Concentration of the soluble form of TLR-2 in serum [ng/mL]	5.62±3.01	5.38 (1.64-15.63)	11.37±7.54	9.14 (1.17-28.46)	0.66

The concentration of the soluble form of TLR-3 in serum [ng/mL]	6.11±0.20	6.12 (5.80-6.55)	9.98±2.56	8.95 (7.48-17.48)	0.000*
The concentration of the soluble form of TLR-4 in serum [ng/mL]	4.86±1.67	4.11 (2.54-10.36)	10.85±4.78	8.72 (4.03-23.57)	0.11
The concentration of the soluble form of TLR-7 in serum [ng/mL]	4.62±0.29	4.52 (4.24-5.30)	7.96±2.32	7.34 (5.71-14.86)	0.000*
The concentration of the soluble form of TLR-8 in serum [ng/mL]	5.82±1.17	5.52 (4.16-9.42)	10.06±2.09	9.54 (5.99-17.86)	0.04*
The concentration of the soluble form of TLR-9 in serum [ng/mL]	7.61±4.30	6.17 (2.53-16.09)	14.62±8.06	14.07 (1.30-31.70)	0.09

Supplementary Materials Table S7. Selected parameters of morphology, biochemistry and immunophenotype of NSCLC patients after four years of observation, taking into account their survival status

Parameters	Patients with NSCLC alive		Patients with NSCLC dead		p-value
	Mean±SD	Median (Range)	Mean±SD	Median (Range)	
WBC [10^3/mm^3]	8.04± 1.90	7.88 (4.78-11.13)	9.95± 3.25	9.25 (5.26-24.73)	0.000*
LYM [10^3/mm^3]	1.84± 0.72	1.64 (0.90-3.60)	1.79± 0.78	1.62 (0.44-4.60)	0.78
MON [10^3/mm^3]	0.68± 0.19	0.66 (0.30-1.17)	0.82± 0.30	0.76 (0.41-1.74)	0.02*
NEU [10^3/mm^3]	5.29± 1.57	5.38 (2.79-8.39)	7.06± 2.96	6.39 (3.48-21.70)	0.000*
RBC [10^6/mm^3]	4.12± 0.46	4.25 (2.87-5.17)	4.57± 0.47	4.67 (3.60-5.36)	0.000*
HGB [g/dl]	12.25± 1.79	12.09 (8.70-16.64)	13.17± 1.58	13.30 (9.20-16.70)	0.000*
PLT [10^3/mm^3]	281.40±99.83	266.22 (126.15-491.00)	339.32±112.85	325.00 (124.00-674.00)	0.000*
CRP	11.97±13.21	6.64 (0.64-56.07)	30.47±31.64	21.54 (0.73-134.43)	0.000*
CD3+ T cells [%]	63.39±8.51	61.58 (52.21-80.02)	68.37±12.06	70.12 (20.40-85.44)	0.000*
CD3+CD8+ T cells [%]	27.16±7.96	27.29 (14.20-45.08)	29.83±11.70	29.82 (8.95-58.72)	0.31
CD3+CD4+ T cells [%]	35.63±7.61	35.44 (22.44-52.01)	38.85±9.79	39.66 (10.37-57.00)	0.08

The ratio of CD3+CD4+ T cells to CD3+CD8+ T cells	1.30±0.51	1.19 (0.58-5.58)	1.58±0.93	1.33 (0.46-5.58)	0.289
CD19+ B cells [%]	6.89±3.14	6.68 (1.44-15.83)	8.35±4.98	7.34 (1.92-21.41)	0.46

Supplementary Materials Table S8. Analysis of the percentage of TLRs tested on selected subpopulations of peripheral blood lymphocytes and their serum concentration in NSCLC patients after four years of observation, taking into account their survival status

Parameters	Patients with NSCLC alive		Patients with NSCLC dead		p-value
	Mean±SD	Median (Range)	Mean±SD	Median (Range)	
CD4+TLR-2+ T cells [%]	2.78±0.91	2.60 (1.26-4.88)	7.11±4.32	5.66 (2.10-21.02)	0.000*
CD8+TLR-2+ T cells [%]	5.72±3.53	4.36 (2.08-13.61)	9.02±4.38	8.33 (3.17-21.93)	0.000*
CD19+TLR-2+ B cells [%]	5.99±3.39	4.80 (1.25-13.20)	9.16±4.46	7.01 (3.96-18.08)	0.000*
CD4+TLR-3+ T cells [%]	1.78±0.48	1.67 (1.02-2.76)	3.08±0.83	2.81 (1.95-4.91)	0.000*
CD8+TLR-3+ T cells [%]	1.50±0.44	1.67 (0.74-2.28)	2.82±0.54	2.77 (1.64-3.98)	0.000*
CD19+TLR-3+ B cells [%]	1.65±0.36	1.82 (0.95-2.29)	3.21±0.60	3.19 (2.12-4.41)	0.000*
CD4+TLR-4+ T cells [%]	5.48±2.77	4.73 (2.30-11.59)	7.91±3.50	6.72 (3.62-17.39)	0.000*
CD8+TLR-4+ T cells [%]	5.46±2.18	5.66 (2.03-9.94)	9.54±4.15	8.06 (3.63-18.80)	0.000*
CD19+TLR-4+ B cells [%]	5.76±2.25	4.90 (3.14-11.34)	8.62±4.79	6.82 (3.79-25.13)	0.000*
CD4+TLR-7+ T cells [%]	1.24±0.21	1.25 (0.97-1.65)	2.24±0.44	2.19 (1.42-3.48)	0.000*
CD8+TLR-7+ T cells [%]	1.58±0.40	1.61 (0.92-2.84)	2.90±0.61	2.79 (1.22-4.47)	0.000*
CD19+TLR-7+ B cells [%]	1.98±0.19	1.99 (1.29-2.38)	3.16±0.48	3.22 (2.24-4.01)	0.000*
CD4+TLR-8+ T cells [%]	2.54±0.31	2.44 (2.10-3.47)	4.66±0.86	4.76 (2.96-6.43)	0.000*
CD8+TLR-8+ T cells [%]	0.99±0.39	1.04 (0.13-1.71)	2.60±0.75	2.40 (1.30-4.08)	0.000*
CD19+TLR-8+ B cells [%]	1.23±0.31	1.07 (0.96-2.01)	2.85±0.75	2.83 (1.20-4.28)	0.000*
CD4+TLR-9+ T cells [%]	5.00±2.67	5.15 (1.90-15.45)	8.16±4.84	7.16 (2.60-21.30)	0.000*
CD8+TLR-9+ T cells [%]	5.57±2.54	5.56 (1.03-9.87)	9.48±4.04	8.44 (3.79-20.59)	0.000*

CD19+TLR-9+ B cells [%]	5.99±3.74	4.67 (1.51-18.50)	14.18±8.37	11.15 (5.52-38.17)	0.000*
Concentration of the soluble form of TLR-2 in serum [ng/mL]	6.09±3.59	5.67 (1.76-18.14)	11.18±7.47	8.51 (1.17-28.46)	0.000*
The concentration of the soluble form of TLR-3 in serum [ng/mL]	6.60±0.20	6.62 (6.25-7.04)	9.88±2.56	8.91 (7.48-17.48)	0.000*
The concentration of the soluble form of TLR-4 in serum [ng/mL]	5.17±1.80	4.44 (2.73-11.12)	10.67±4.79	8.63 (4.03-23.57)	0.000*
Concentration of the soluble form of TLR-7 in serum [ng/mL]	4.96±0.27	4.90 (4.57-5.69)	7.88±2.31	7.24 (5.71-14.86)	0.000*
Concentration of the soluble form of TLR-8 in serum [ng/mL]	6.21±1.26	5.84 (4.46-10.12)	9.97±2.10	9.47 (5.99-17.86)	0.000*
Concentration of the soluble form of TLR-9 in serum [ng/mL]	6.21±1.26	5.84 (4.46-10.12)	14.59±7.95	14.04 (1.30-31.70)	0.000*

Supplementary Materials Table S9. Spearman rank correlation analysis for dead NSCLC patients

Parameter	R Spearman	t(N-2)	p-value	Parameter	R Spearman	t(N-2)	p-value
CD8+TLR-2+ [%] & CD8+TLR-9+ [%]	0.404	3.748	0.000*	CD8+TLR-2+ [%] & CD19+TLR-2+ [%]	0.655	7.351	0.000*
CD8+TLR-7+ [%] & sTLR-4 [ng/mL]	0.410	3.816	0.000*	CD19+TLR-2+ [%] & sTLR-9 [ng/mL]	0.659	7.431	0.000*
CD19+TLR-4+ [%] & CD4+TLR-7+ [%]	0.411	3.826	0.000*	CD8+TLR-4+ [%] & CD19+TLR-9+ [%]	0.659	7.436	0.000*
CD8+TLR-2+ [%] & CD19+TLR-9+ [%]	0.419	3.914	0.000*	CD8+TLR-9+ [%] & sTLR-4 [ng/mL]	0.660	7.450	0.000*
CD19+TLR-4+ [%] & CD4+TLR-8+ [%]	0.425	3.982	0.000*	CD8+TLR-7+ [%] & sTLR-7 [ng/mL]	0.667	7.603	0.000*
CD4+TLR-3+ [%] & CD8+TLR-4+ [%]	0.425	3.986	0.000*	CD4+TLR-4+ [%] & CD8+TLR-4+ [%]	0.668	7.625	0.000*
CD4+TLR-3+ [%] & sTLR-4 [ng/mL]	0.429	4.029	0.000*	CD4+TLR-4+ [%] & sTLR-4 [ng/mL]	0.676	7.775	0.000*
CD19+TLR-3+ [%] & sTLR-4 [ng/mL]	0.431	4.052	0.000*	CD19+TLR-2+ [%] & sTLR-4 [ng/mL]	0.678	7.823	0.000*
CD19+TLR-7+ [%] & sTLR-4 [ng/mL]	0.432	4.065	0.000*	CD19+TLR-8+ [%] & sTLR-8 [ng/mL]	0.678	7.834	0.000*
CD19+TLR-2+ [%] & CD8+TLR-7+ [%]	0.435	4.100	0.000*	CD19+TLR-9+ [%] & sTLR-4 [ng/mL]	0.680	7.874	0.000*
CD19+TLR-2+ [%] & CD4+TLR-7+ [%]	0.437	4.122	0.000*	CD8+TLR-2+ [%] & CD4+TLR-8+ [%]	0.681	7.885	0.000*
CD19+TLR-2+ [%] & sTLR-7 [ng/mL]	0.438	4.130	0.000*	CD19+TLR-2+ [%] & sTLR-2 [ng/mL]	0.691	8.109	0.000*
CD19+TLR-2+ [%] & CD19+TLR-8+ [%]	0.440	4.157	0.000*	CD8+TLR-2+ [%] & CD8+TLR-7+ [%]	0.695	8.199	0.000*
CD19+TLR-4+ [%] & CD8+TLR-7+ [%]	0.440	4.159	0.000*	CD8+TLR-2+ [%] & sTLR-7 [ng/mL]	0.709	8.524	0.000*

CD4+TLR-4+ [%] & CD19+TLR-8+ [%]	0.441	4.171	0.000*	CD8+TLR-2+ [%] & CD4+TLR-4+ [%]	0.710	8.555	0.000*
CD4+TLR-4+ [%] & CD4+TLR-7+ [%]	0.444	4.201	0.000*	CD4+TLR-3+ [%] & CD19+TLR-8+ [%]	0.714	8.644	0.000*
CD19+TLR-2+ [%] & CD4+TLR-8+ [%]	0.444	4.205	0.000*	CD8+TLR-2+ [%] & CD8+TLR-8+ [%]	0.717	8.719	0.000*
CD19+TLR-2+ [%] & sTLR-8 [ng/mL]	0.445	4.211	0.000*	CD8+TLR-2+ [%] & CD19+TLR-7+ [%]	0.717	8.726	0.000*
CD8+TLR-3+ [%] & sTLR-8 [ng/mL]	0.446	4.225	0.000*	CD8+TLR-2+ [%] & sTLR-3 [ng/mL]	0.718	8.741	0.000*
CD19+TLR-4+ [%] & CD8+TLR-8+ [%]	0.447	4.237	0.000*	CD8+TLR-2+ [%] & CD19+TLR-3+ [%]	0.718	8.741	0.000*
CD4+TLR-4+ [%] & sTLR-7 [ng/mL]	0.448	4.247	0.000*	CD4+TLR-2+ [%] & sTLR-8 [ng/mL]	0.723	8.869	0.000*
CD8+TLR-8+ [%] & sTLR-4 [ng/mL]	0.448	4.247	0.000*	CD4+TLR-3+ [%] & CD4+TLR-7+ [%]	0.724	8.894	0.000*
CD4+TLR-4+ [%] & CD19+TLR-4+ [%]	0.453	4.310	0.000*	CD19+TLR-2+ [%] & CD4+TLR-4+ [%]	0.729	9.028	0.000*
CD4+TLR-3+ [%] & CD8+TLR-3+ [%]	0.454	4.325	0.000*	CD8+TLR-4+ [%] & CD8+TLR-9+ [%]	0.731	9.080	0.000*
CD4+TLR-4+ [%] & CD19+TLR-9+ [%]	0.459	4.384	0.000*	CD19+TLR-9+ [%] & sTLR-2 [ng/mL]	0.733	9.138	0.000*
CD8+TLR-2+ [%] & sTLR-2 [ng/mL]	0.459	4.388	0.000*	CD4+TLR-8+ [%] & sTLR-8 [ng/mL]	0.737	9.249	0.000*
CD4+TLR-3+ [%] & CD8+TLR-9+ [%]	0.461	4.412	0.000*	CD4+TLR-7+ [%] & sTLR-8 [ng/mL]	0.747	9.547	0.000*
CD8+TLR-2+ [%] & CD19+TLR-4+ [%]	0.464	4.440	0.000*	CD4+TLR-3+ [%] & CD4+TLR-8+ [%]	0.749	9.587	0.000*
CD19+TLR-3+ [%] & CD19+TLR-4+ [%]	0.464	4.442	0.000*	CD4+TLR-9+ [%] & sTLR-4 [ng/mL]	0.749	9.604	0.000*
CD19+TLR-4+ [%] & sTLR-7 [ng/mL]	0.464	4.447	0.000*	CD8+TLR-4+ [%] & sTLR-9 [ng/mL]	0.758	9.865	0.000*
CD19+TLR-4+ [%] & CD19+TLR-7+ [%]	0.464	4.450	0.000*	CD4+TLR-3+ [%] & CD8+TLR-8+ [%]	0.764	10.057	0.000*
CD19+TLR-4+ [%] & sTLR-3 [ng/mL]	0.466	4.468	0.000*	CD4+TLR-2+ [%] & CD19+TLR-8+ [%]	0.766	10.098	0.000*
CD4+TLR-4+ [%] & CD4+TLR-8+ [%]	0.472	4.546	0.000*	CD4+TLR-3+ [%] & sTLR-7 [ng/mL]	0.766	10.102	0.000*
CD4+TLR-3+ [%] & CD4+TLR-4+ [%]	0.475	4.582	0.000*	CD8+TLR-4+ [%] & CD4+TLR-9+ [%]	0.774	10.368	0.000*
CD4+TLR-2+ [%] & sTLR-4 [ng/mL]	0.482	4.669	0.000*	CD19+TLR-3+ [%] & sTLR-8 [ng/mL]	0.774	10.369	0.000*
CD19+TLR-2+ [%] & CD8+TLR-8+ [%]	0.483	4.678	0.000*	CD19+TLR-7+ [%] & sTLR-8 [ng/mL]	0.774	10.389	0.000*
CD4+TLR-2+ [%] & CD19+TLR-2+ [%]	0.483	4.682	0.000*	CD8+TLR-8+ [%] & sTLR-8 [ng/mL]	0.775	10.419	0.000*
CD19+TLR-2+ [%] & CD19+TLR-3+ [%]	0.484	4.694	0.000*	CD4+TLR-3+ [%] & sTLR-3 [ng/mL]	0.781	10.616	0.000*
CD19+TLR-2+ [%] & sTLR-3 [ng/mL]	0.485	4.704	0.000*	CD4+TLR-3+ [%] & CD19+TLR-3+ [%]	0.781	10.624	0.000*
CD19+TLR-2+ [%] & CD19+TLR-7+ [%]	0.485	4.705	0.000*	CD4+TLR-3+ [%] & CD19+TLR-7+ [%]	0.782	10.649	0.000*
CD19+TLR-2+ [%] & CD4+TLR-3+ [%]	0.489	4.760	0.000*	CD8+TLR-9+ [%] & sTLR-2 [ng/mL]	0.802	11.398	0.000*
CD4+TLR-2+ [%] & CD19+TLR-4+ [%]	0.493	4.805	0.000*	CD8+TLR-9+ [%] & CD19+TLR-9+ [%]	0.806	11.556	0.000*

CD8+TLR-7+ [%] & sTLR-8 [ng/mL]	0.494	4.824	0.000*	CD4+TLR-2+ [%] & CD4+TLR-7+ [%]	0.812	11.797	0.000*
CD19+TLR-3+ [%] & CD4+TLR-4+ [%]	0.498	4.873	0.000*	CD4+TLR-2+ [%] & CD8+TLR-2+ [%]	0.812	11.798	0.000*
CD4+TLR-4+ [%] & CD19+TLR-7+ [%]	0.498	4.874	0.000*	CD19+TLR-9+ [%] & sTLR-9 [ng/mL]	0.813	11.863	0.000*
CD4+TLR-4+ [%] & sTLR-3 [ng/mL]	0.499	4.888	0.000*	CD8+TLR-4+ [%] & sTLR-2 [ng/mL]	0.816	11.957	0.000*
CD4+TLR-4+ [%] & CD8+TLR-8+ [%]	0.504	4.958	0.000*	CD4+TLR-9+ [%] & sTLR-2 [ng/mL]	0.816	11.989	0.000*
CD8+TLR-2+ [%] & CD8+TLR-4+ [%]	0.510	5.035	0.000*	CD4+TLR-2+ [%] & sTLR-7 [ng/mL]	0.830	12.603	0.000*
CD8+TLR-2+ [%] & CD8+TLR-3+ [%]	0.514	5.090	0.000*	CD4+TLR-2+ [%] & CD4+TLR-8+ [%]	0.830	12.625	0.000*
CD4+TLR-4+ [%] & sTLR-9 [ng/mL]	0.519	5.157	0.000*	CD4+TLR-9+ [%] & CD8+TLR-9+ [%]	0.833	12.799	0.000*
CD4+TLR-4+ [%] & CD8+TLR-7+ [%]	0.521	5.184	0.000*	CD8+TLR-4+ [%] & sTLR-4 [ng/mL]	0.837	12.968	0.000*
CD19+TLR-2+ [%] & CD19+TLR-9+ [%]	0.523	5.206	0.000*	CD4+TLR-9+ [%] & CD19+TLR-9+ [%]	0.840	13.163	0.000*
CD8+TLR-3+ [%] & CD8+TLR-7+ [%]	0.539	5.435	0.000*	CD8+TLR-9+ [%] & sTLR-9 [ng/mL]	0.845	13.415	0.000*
CD4+TLR-7+ [%] & CD8+TLR-7+ [%]	0.540	5.437	0.000*	CD19+TLR-8+ [%] & sTLR-7 [ng/mL]	0.856	14.027	0.000*
CD4+TLR-4+ [%] & CD8+TLR-9+ [%]	0.544	5.502	0.000*	CD4+TLR-2+ [%] & CD19+TLR-7+ [%]	0.865	14.625	0.000*
CD8+TLR-2+ [%] & CD4+TLR-3+ [%]	0.546	5.529	0.000*	CD4+TLR-2+ [%] & CD19+TLR-3+ [%]	0.865	14.658	0.000*
CD8+TLR-3+ [%] & CD19+TLR-8+ [%]	0.556	5.680	0.000*	CD4+TLR-2+ [%] & sTLR-3 [ng/mL]	0.865	14.661	0.000*
CD8+TLR-2+ [%] & sTLR-8 [ng/mL]	0.559	5.721	0.000*	CD4+TLR-7+ [%] & CD19+TLR-8+ [%]	0.869	14.880	0.000*
CD4+TLR-2+ [%] & CD4+TLR-4+ [%]	0.559	5.727	0.000*	CD4+TLR-2+ [%] & CD8+TLR-8+ [%]	0.874	15.270	0.000*
CD8+TLR-7+ [%] & CD19+TLR-8+ [%]	0.573	5.926	0.000*	CD4+TLR-9+ [%] & sTLR-9 [ng/mL]	0.887	16.297	0.000*
CD4+TLR-4+ [%] & CD4+TLR-9+ [%]	0.576	5.977	0.000*	CD8+TLR-8+ [%] & CD19+TLR-8+ [%]	0.889	16.492	0.000*
CD8+TLR-3+ [%] & CD4+TLR-7+ [%]	0.582	6.071	0.000*	CD19+TLR-7+ [%] & CD19+TLR-8+ [%]	0.902	17.741	0.000*
CD19+TLR-2+ [%] & CD8+TLR-9+ [%]	0.589	6.191	0.000*	CD19+TLR-8+ [%] & sTLR-3 [ng/mL]	0.902	17.744	0.000*
CD4+TLR-2+ [%] & CD8+TLR-3+ [%]	0.597	6.316	0.000*	CD19+TLR-3+ [%] & CD19+TLR-8+ [%]	0.903	17.860	0.000*
CD8+TLR-7+ [%] & CD4+TLR-8+ [%]	0.598	6.328	0.000*	CD4+TLR-8+ [%] & CD19+TLR-8+ [%]	0.909	18.534	0.000*
CD4+TLR-3+ [%] & sTLR-8 [ng/mL]	0.599	6.348	0.000*	CD4+TLR-7+ [%] & sTLR-7 [ng/mL]	0.909	18.565	0.000*
CD4+TLR-3+ [%] & CD8+TLR-7+ [%]	0.602	6.402	0.000*	CD4+TLR-8+ [%] & sTLR-7 [ng/mL]	0.918	19.665	0.000*
CD4+TLR-4+ [%] & sTLR-2 [ng/mL]	0.608	6.497	0.000*	CD8+TLR-8+ [%] & sTLR-7 [ng/mL]	0.944	24.160	0.000*
CD8+TLR-3+ [%] & sTLR-7 [ng/mL]	0.609	6.517	0.000*	CD4+TLR-7+ [%] & CD8+TLR-8+ [%]	0.944	24.300	0.000*
CD19+TLR-2+ [%] & CD4+TLR-9+ [%]	0.611	6.554	0.000*	CD19+TLR-7+ [%] & sTLR-7 [ng/mL]	0.951	26.241	0.000*

CD8+TLR-3+ [%] & CD4+TLR-8+ [%]	0.618	6.668	0.000*	CD4+TLR-7+ [%] & sTLR-3 [ng/mL]	0.952	26.297	0.000*
CD8+TLR-3+ [%] & CD8+TLR-8+ [%]	0.627	6.832	0.000*	CD4+TLR-7+ [%] & CD19+TLR-7+ [%]	0.952	26.300	0.000*
CD8+TLR-7+ [%] & CD8+TLR-8+ [%]	0.627	6.834	0.000*	CD19+TLR-3+ [%] & sTLR-7 [ng/mL]	0.952	26.351	0.000*
CD8+TLR-3+ [%] & CD19+TLR-7+ [%]	0.627	6.835	0.000*	CD19+TLR-3+ [%] & CD4+TLR-7+ [%]	0.952	26.459	0.000*
CD8+TLR-3+ [%] & sTLR-3 [ng/mL]	0.628	6.840	0.000*	CD4+TLR-8+ [%] & CD8+TLR-8+ [%]	0.958	28.199	0.000*
CD4+TLR-2+ [%] & CD8+TLR-7+ [%]	0.628	6.851	0.000*	CD19+TLR-7+ [%] & CD4+TLR-8+ [%]	0.966	31.943	0.000*
CD8+TLR-2+ [%] & sTLR-4 [ng/mL]	0.629	6.870	0.000*	CD4+TLR-8+ [%] & sTLR-3 [ng/mL]	0.966	31.944	0.000*
CD8+TLR-3+ [%] & CD19+TLR-3+ [%]	0.630	6.883	0.000*	CD19+TLR-3+ [%] & CD4+TLR-8+ [%]	0.967	32.274	0.000*
CD4+TLR-2+ [%] & CD4+TLR-3+ [%]	0.632	6.925	0.000*	CD4+TLR-7+ [%] & CD4+TLR-8+ [%]	0.972	35.248	0.000*
CD19+TLR-3+ [%] & CD8+TLR-7+ [%]	0.637	7.013	0.000*	CD19+TLR-3+ [%] & CD8+TLR-8+ [%]	0.991	63.032	0.000*
CD8+TLR-7+ [%] & CD19+TLR-7+ [%]	0.637	7.015	0.000*	CD19+TLR-7+ [%] & CD8+TLR-8+ [%]	0.991	63.461	0.000*
CD8+TLR-7+ [%] & sTLR-3 [ng/mL]	0.637	7.015	0.000*	CD8+TLR-8+ [%] & sTLR-3 [ng/mL]	0.991	63.651	0.000*
CD8+TLR-2+ [%] & CD19+TLR-8+ [%]	0.639	7.042	0.000*	CD19+TLR-3+ [%] & sTLR-3 [ng/mL]	1.000	409.343	0.000*
CD8+TLR-2+ [%] & CD4+TLR-7+ [%]	0.642	7.111	0.000*	CD19+TLR-3+ [%] & CD19+TLR-7+ [%]	1.000	449.978	0.000*
CD19+TLR-2+ [%] & CD8+TLR-4+ [%]	0.654	7.328	0.000*	CD19+TLR-7+ [%] & sTLR-3 [ng/mL]	1.000	481.066	0.000*

* statistically significant results

Supplementary Materials Table S10. Spearman rank correlation analysis for alive NSCLC patients

Parameter	R Spearman	t(N-2)	p-value	Parameter	R Spearman	t(N-2)	p-value
CD8+TLR-8+ [%] & sTLR-2 [ng/mL]	-0.821	-5.384	0.000*	CD4+TLR-9+ [%] & CD8+TLR-2+ [%]	0.635	3.078	0.008*
CD4+TLR-4+ [%] & sTLR-2 [ng/mL]	-0.771	-4.524	0.000*	CD4+TLR-9+ [%] & CD8+TLR-7+ [%]	0.643	3.142	0.007*
CD19+TLR-8+ [%] & sTLR-2 [ng/mL]	-0.729	-3.985	0.001*	CD4+TLR-4+ [%] & CD8+TLR-2+ [%]	0.650	3.200	0.006*
CD19+TLR-7+ [%] & sTLR-2 [ng/mL]	-0.722	-3.903	0.002*	CD19+TLR-4+ [%] & CD4+TLR-4+ [%]	0.650	3.200	0.006*
CD4+TLR-7+ [%] & sTLR-2 [ng/mL]	-0.704	-3.709	0.002*	CD8+TLR-8+ [%] & CD4+TLR-4+ [%]	0.664	3.320	0.005*
CD4+TLR-8+ [%] & sTLR-2 [ng/mL]	-0.704	-3.709	0.002*	CD4+TLR-4+ [%] & CD19+TLR-2+ [%]	0.665	3.329	0.005*
CD19+TLR-3+ [%] & sTLR-2 [ng/mL]	-0.660	-3.290	0.005*	CD19+TLR-8+ [%] & CD4+TLR-4+ [%]	0.667	3.351	0.005*
CD4+TLR-4+ [%] & sTLR-4 [ng/mL]	-0.553	-2.483	0.026*	CD8+TLR-4+ [%] & CD8+TLR-2+ [%]	0.668	3.356	0.005*
CD4+TLR-2+ [%] & CD4+TLR-4+ [%]	-0.531	-2.346	0.034*	CD8+TLR-7+ [%] & CD19+TLR-2+ [%]	0.687	3.540	0.003*

CD19+TLR-4+ [%] & sTLR-2 [ng/mL]	-0.529	-2.335	0.035*	CD19+TLR-9+ [%] & CD8+TLR-9+ [%]	0.692	3.589	0.003*
CD19+TLR-4+ [%] & CD4+TLR-2+ [%]	-0.506	-2.197	0.045*	CD19+TLR-2+ [%] & CD8+TLR-2+ [%]	0.697	3.638	0.003*
CD19+TLR-3+ [%] & CD4+TLR-8+ [%]	0.500	2.162	0.048*	CD4+TLR-9+ [%] & CD19+TLR-2+ [%]	0.703	3.698	0.002*
CD4+TLR-8+ [%] & CD19+TLR-3+ [%]	0.500	2.162	0.048*	CD4+TLR-9+ [%] & CD4+TLR-4+ [%]	0.712	3.791	0.002*
CD19+TLR-3+ [%] & sTLR-3 [ng/mL]	0.501	2.165	0.048*	CD8+TLR-9+ [%] & CD8+TLR-2+ [%]	0.726	3.955	0.001*
CD19+TLR-4+ [%] & sTLR-7 [ng/mL]	0.506	2.192	0.046*	CD8+TLR-9+ [%] & CD4+TLR-9+ [%]	0.729	3.990	0.001*
CD19+TLR-7+ [%] & CD19+TLR-4+ [%]	0.513	2.238	0.042*	CD19+TLR-9+ [%] & CD8+TLR-7+ [%]	0.749	4.226	0.001*
CD19+TLR-9+ [%] & CD8+TLR-2+ [%]	0.515	2.251	0.041*	CD4+TLR-8+ [%] & CD8+TLR-8+ [%]	0.803	5.033	0.000*
CD19+TLR-7+ [%] & CD19+TLR-3+ [%]	0.517	2.262	0.040*	CD8+TLR-8+ [%] & CD4+TLR-8+ [%]	0.803	5.033	0.000*
CD8+TLR-8+ [%] & CD19+TLR-4+ [%]	0.519	2.275	0.039*	CD8+TLR-7+ [%] & CD8+TLR-2+ [%]	0.806	5.104	0.000*
CD19+TLR-3+ [%] & sTLR-7 [ng/mL]	0.521	2.286	0.038*	CD8+TLR-8+ [%] & sTLR-3 [ng/mL]	0.807	5.114	0.000*
CD19+TLR-9+ [%] & CD19+TLR-4+ [%]	0.532	2.349	0.034*	CD8+TLR-8+ [%] & CD19+TLR-7+ [%]	0.810	5.164	0.000*
CD19+TLR-4+ [%] & sTLR-3 [ng/mL]	0.534	2.365	0.033*	CD8+TLR-8+ [%] & sTLR-7 [ng/mL]	0.819	5.347	0.000*
CD19+TLR-9+ [%] & CD19+TLR-2+ [%]	0.536	2.376	0.032*	CD19+TLR-4+ [%] & CD19+TLR-2+ [%]	0.838	5.752	0.000*
CD4+TLR-8+ [%] & CD19+TLR-4+ [%]	0.539	2.395	0.031*	CD8+TLR-8+ [%] & CD19+TLR-3+ [%]	0.850	6.045	0.000*
CD4+TLR-7+ [%] & CD19+TLR-4+ [%]	0.543	2.422	0.030*	CD19+TLR-8+ [%] & CD8+TLR-8+ [%]	0.856	6.184	0.000*
CD19+TLR-4+ [%] & CD8+TLR-2+ [%]	0.553	2.483	0.026*	CD8+TLR-9+ [%] & CD8+TLR-7+ [%]	0.857	6.209	0.000*
CD8+TLR-7+ [%] & CD19+TLR-4+ [%]	0.553	2.486	0.026*	CD19+TLR-8+ [%] & CD19+TLR-7+ [%]	0.956	12.129	0.000*
CD8+TLR-4+ [%] & CD4+TLR-4+ [%]	0.562	2.541	0.024*	CD19+TLR-8+ [%] *-7 [ng/mL]	0.962	13.114	0.000*
CD8+TLR-9+ [%] & CD8+TLR-4+ [%]	0.562	2.541	0.024*	CD19+TLR-8+ [%] *& sTLR-3 [ng/mL]	0.962	13.260	0.000*
CD19+TLR-8+ [%] & CD19+TLR-4+ [%]	0.571	2.605	0.021*	CD4+TLR-8+ [%] & *CD19+TLR-8+ [%]	0.968	14.502	0.000*
CD4+TLR-4+ [%] & sTLR-3 [ng/mL]	0.602	2.820	0.014*	CD19+TLR-8+ [%] & CD4+TLR-8+ [%]	0.968	14.502	0.000*
CD4+TLR-4+ [%] & sTLR-7 [ng/mL]	0.603	2.827	0.013*	CD19+TLR-7+ [%] & CD4+TLR-7+ [%]	0.991	27.891	0.000*
CD19+TLR-7+ [%] & CD4+TLR-4+ [%]	0.607	2.854	0.013*	CD19+TLR-7+ [%] & sTLR-3 [ng/mL]	0.992	29.166	0.000*

CD4+TLR-4+ [%] & CD4+TLR-8+ [%]	0.611	2.889	0.012*	CD4+TLR-8+ [%] & CD19+TLR-7+ [%]	0.993	32.265	0.000*
CD4+TLR-8+ [%] & CD4+TLR-4+ [%]	0.611	2.889	0.012*	CD19+TLR-7+ [%] & sTLR-7 [ng/mL]	0.995	36.610	0.000*
CD19+TLR-9+ [%] & CD4+TLR-9+ [%]	0.614	2.912	0.011*	CD4+TLR-7+ [%] & sTLR-7 [ng/mL]	0.995	36.669	0.000*
CD8+TLR-7+ [%] & CD8+TLR-4+ [%]	0.615	2.919	0.011*	CD4+TLR-8+ [%] & sTLR-7 [ng/mL]	0.995	36.669	0.000*
CD8+TLR-9+ [%] & CD19+TLR-2+ [%]	0.624	2.984	0.010*	CD4+TLR-7+ [%] & sTLR-3 [ng/mL]	0.998	56.180	0.000*
CD19+TLR-8+ [%] & CD19+TLR-3+ [%]	0.630	3.037	0.009*	CD4+TLR-8+ [%] & sTLR-3 [ng/mL]	0.998	56.180	0.000*
CD4+TLR-9+ [%] & CD19+TLR-4+ [%]	0.632	3.054	0.009*	CD4+TLR-8+ [%] & CD4+TLR-7+ [%]	0.999	68.815	0.000*

* Statistically significant results
